# Central Med23 deficiency leads to malformation of dentate gyrus and ADHD-like behaviors in mice

**DOI:** 10.1038/s41386-025-02088-1

**Published:** 2025-03-20

**Authors:** Bing-Yao Zhou, Ze-Xuan Li, Yi-Wei Li, Jin-Nan Li, Wei-Tang Liu, Xi-Yue Liu, Zhi-Bin Hu, Li Zhao, Jia-Yin Chen, Ling Hu, Ning-Ning Song, Xue Feng, Gang Wang, Lin Xu, Yu-Qiang Ding

**Affiliations:** 1 State Key Laboratory of Medical Neurobiology and MOE Frontiers Center for Brain Science, Institute of Brain Science, Fudan University, Shanghai, 200032 China; 2 Laboratory Animal Center, Fudan University, Shanghai, 200032 China; 3 Key Laboratory of Animal Models and Human Disease Mechanisms, Kunming Institute of Zoology, the Chinese Academy of Science, Kunming, 650223 China; 4 Shanghai Institute of Infectious Disease and Biosecurity, Fudan University, Shanghai, 200032 China; 5 State Key Laboratory of Genetic Engineering, School of Life Sciences and Zhongshan Hospital, Fudan University, Shanghai, 200438 China; 6 Huashan Institute of Medicine (HS-IOM), Huashan Hospital, Fudan University, Shanghai, 200040 China

**Keywords:** Developmental disorders, ADHD

## Abstract

Attention-deficit hyperactivity disorder (ADHD) is a prevalent psychiatric disorder with high heritability, while its etiology and pathophysiology remain unclear. Med23 is a subunit of the Mediator complex, a key regulator of gene expression by linking transcription factors to RNA polymerase II. The mutations of Med23 are associated with several brain diseases including microcephaly, epilepsy and intellectual disability, but its biological roles in brain development and possible behavioral consequence have not been explored in the animal model. In this study, Emx1-Cre mice were used to generate Med23 conditional knockout (Med23 CKO) mice that showed severe hypoplasia of the dentate gyrus (DG) with malformation of the dendritic tree and spines along with impaired short-term synaptic plasticity. Interestingly, Med23 CKO mice exhibited ADHD-like behaviors as shown by hyperactivity, inattention and impulsivity, as well as impaired sensory gating and working memory. Importantly, methylphenidate (MPH), a common drug for ADHD ameliorated these deficits in the CKO mice. Furthermore, we also revealed that the impaired synaptic plasticity was partially restored by MPH in an N-methyl-d-aspartate (NMDA) receptor-dependent way. Collectively, our data demonstrate Med23 deficiency causes DG malformation and ADHD-like behaviors, suggesting a novel mechanism underlying relevant brain diseases.

## Introduction

Attention-deficit hyperactivity disorder (ADHD) is a common psychiatric disorder with a global prevalence of around 5%, and characterized with persistent and developmentally-inappropriate levels of hyperactivity, inattention and impulsivity [1], along with deficits in sensory gating and working memory [2, 3]. Family, twin, and adoption studies show that ADHD is highly heritable with heritability up to 74%, which leads to genetic factors playing fundamental roles in the pathogenesis of ADHD [4]. Multiple studies have observed a strong genetic association with the changes in brain structures and functions, as well as abnormal and delayed neurodevelopment in ADHD [5]. Brain imaging studies in patients with ADHD have reported significant structural and functional differences in whole brain volume and several brain regions, including the hippocampus [6, 7].

The hippocampal formation is comprised of CA1-CA3 subregions, dentate gyrus (DG), subicular complex and entorhinal cortex (EC) [8]. The DG receives mass highly-processed information input from the EC and sends integrated output to CA regions to participate in various brain functions such as learning, memory and sensorimotor integration [9, 10]. Reports are emerging of hippocampal involvement in the development of ADHD based on the fact that the functions of the hippocampus are closely related to the deficits in cognitive and executive ability in ADHD patients leading to poor academic performance and social behaviors [11, 12]. Reduced volume, abnormal morphology and altered functional connectivity within the hippocampus in varying degrees were observed as well [7, 13, 14]. However, more studies are needed to further elucidate the potential role of the hippocampus in the pathogenesis of ADHD.

Med23 is a component in the tail part of the Mediator complex, which is a highly-conserved multiprotein complex that takes an essential role in regulating the spatiotemporal gene expression [15]. Previous studies have revealed the roles of Med23 in multiple physiological processes, including lung tumorigenesis, muscle regeneration, angiogenesis, and neointima formation [16–18]. On the other hand, fewer studies are available concerning exact biological functions of Med23 in the central nervous system. Interestingly, clinical genetic evidence has linked Med23 mutation or down-regulated expression to intellectual disability, refractory epilepsy and microcephaly [19–21], suggesting possible involvement in brain-development diseases. Besides, Med23 has been reported to be associated with ADHD that the whole blood Med23 RNA level is significantly reduced in ADHD patients [22].

In this study, we generated Med23 CKO mice by crossing Med23^flox/flox^ mice with Emx1-Cre mice, in which Med23 was deleted in the cerebral cortex and hippocampus at embryonic stage. The CKO mice survived to adulthood without gross abnormality. Brain morphological examination showed a severe malformation of the hippocampus, as shown by much reduced DG volume but intact CA regions. Critically, the CKO mice showed the dysfunction of EC-DG neural circuit and ADHD-like behavioral phenotypes, which were ameliorated by MPH, a clinically prescribed medication to treat ADHD. Our results provided biological evidence for understanding Med23 deficiency-mediated ADHD-like disease.

## Materials and methods

### Sex as a biological variable

Both male and female mice (balanced for sex) were used in our study for all the behavioral and biochemical experiments with similar findings reported for both sexes (Supplementary Fig. 1). Therefore, in this study, sex was not considered a biological variable.

### Animals

Med23^flox/flox^ mice with Med23 exons 5–7 allele flanked by LoxP sites were described in our previous work [23]. By crossing Emx1-Cre mice [24] with Med23^flox/flox^ mice, we generated Med23 CKO (Emx1-Cre:Med23^flox/flox^) mice for research. Littermates of other genotypes (i.e., Med23^flox/+^, Med23^flox/flox^, Emx1-Cre and wild type) were all used as controls. The mouse strains used in this study were kept in specific pathogen–free (SPF) conditions, and maintained on C57BL/6 J background.

### Tissue preparation

Mice received a deep anesthesia with sodium pentobarbital dissolved in saline (40 mg/kg body weight) and then intracardially perfused with 4% paraformaldehyde (PFA) in phosphate-buffered saline (PBS). Brains were removed, postfixed in 4% PFA at 4 °C overnight, cryoprotected in 25% sucrose in PBS overnight and cut into 25 μm-thick coronal sections.

### Nissl staining and immunohistochemistry

For Nissl staining, the sections were washed in PBS, stained with 1% crystal violet, followed by incubation in 95% and 100% ethanol, and xylene. Finally, the sections were mounted in neutral balsam with coverslips.

For immunostaining, the sections were incubated in antigen retrieval buffers (10 mM sodium citrate, 0.05% tween-20, pH 6.0) at 95 °C for 15 min. Then the sections were transferred into blocking serum (1% fetal bovine serum, 0.5% Triton X-100) for 1 h at room temperature and incubated with primary antibodies at 4 °C overnight. After being washed in PBS, the sections were stained with secondary antibodies (Alexa488-conjugated or Biotin-conjugated) at room temperature for 2 h. For those stained with biotin-conjugated secondary antibodies, 1-h incubation of cy3-conjugated streptavidin was applied. All slices were counterstained with Hoechst 33258 (Sigma, 94403, 1:2000) and then mounted in 75% glycerol with coverslips. The following primary antibodies were listed: anti-Ctip2 (Abcam, AB18465, 1:500).

### Golgi staining

Golgi staining was carried out with FD Rapid GolgiStain Kit (PK401, FD NeuroTechnologies, USA) according to the manufacturer’s protocols. Solutions A and B needed to be mixed 24 h before use. PFA-fixed brains were placed in the premixed solution and refreshed the solution on the next day. After 2 weeks of incubation in the darkness, brains were transferred into solution C for 2–7 days, and finally were sectioned into 100-μm-thick slices. The secondary dendritic branches of the granule cells in the DG were selected to analyze spine density and the proportion of different categories with four spine shapes.

### Western blot

The hippocampal tissues were lysed in precooled RIPA buffer containing 1% Triton-X 100, and protease inhibitor cocktails (ab2011111, abcam, UK). Samples were then boiled at 100 °C for 5 min to prevent protein denaturation. After cooling to room temperature, samples were loaded on SDS-PAGE and then transferred to a membrane filter, followed by incubating with 5% non-fat milk in TBST for 2 h at room temperature. After rinsing once with TBST, primary antibodies were applied overnight at 4 °C. All the antibodies were visualized by an ECL kit (34578, Thermo Fisher Scientific, USA), following incubating with HRP-conjugated anti-rabbit IgG (Proteintech, SA00001-2, 1:2000) for 2 h at room temperature. Primary antibodies used for western blot in this study were listed: anti-DRIP130/Med23 (Abcam, ab200351, 1:1000) and anti-GAPDH (Proteintech, 81640-5-RR, 1:3000).

### RT-qPCR

Total RNA was extracted from the hippocampus with RNAiso Plus following the manufacture’s protocol (9109, TaKaRa Biotechnology, Japan) and then the PrimeScript™ RT reagent Kit (TakaRa_RR037A, TaKaRa Biotechnology, Japan) was used to generate cDNA. Each sample was triplicated in RT-qPCR using ABI-Q7 (Applied Biosystems, USA) with RT² SYBR Green ROX qPCR Mastermix (330524, Qiagen, Germany). The sequences of primers are listed:

Med23-F:3ʹ-TCGGAAAATCATTGGAGGAG-5ʹ;

Med23-R:3ʹ-CAATAGGCAGGCATTTCGTT-5ʹ;

GAPDH-F:3ʹ-AACTTTGGCATTGTGGAAGG-5ʹ;

GAPDH-R:3ʹ-ACACATTGGGGGTAGGAACA-5ʹ.

### Behavioral tests

Adult (3–6 months old) mice were used in the following behavioral tests. Behavioral experiments were all performed during the light phase of the 12 h light/dark cycle, and behavioral tests were carried out in sound-proof rooms with a neutral environment. Mice were transported to the experiment room 30 min beforehand for habituation, and behavioral tests included the open field test, object-based attention test, elevated plus maze, Y maze, cliff avoidance reaction (CAR) and pre-pulse inhibition test. The experimenter was blind to the genotypes of mice in the tests.

### Open field test

The open field apparatus comprised a square arena with walls around that are all made of white acrylic plates (40 × 40 ×  40 cm, L × W × H). The activities of mice in the apparatus were captured and analyzed automatically by computer (Omnitech SuperFlex, Omnitech Electronics Inc, USA). The center square of 20 ×  20 cm was set as the center zone while the other area was the peripheral zone. Mice were allowed to explore the apparatus freely for 30 min. Total traveling distance, distance traveling per 10 min, ambulatory time, average velocity, times spent in center or peripheral zone and vertical or stereotypic activity were recorded.

### Y maze

Spontaneous alternation behavior (SAB) in the Y maze test requires attention and working memory [25]. During the tests, mice were placed individually at the end of an arm and allowed to explore the maze freely for 10 min. The order of arm entry and total number of arm entries were recorded manually. An arm entry was defined as the center of the body into one arm. The actual alternations (successive entries into the 3 different arms of the Y-maze without overlapping) and the maximum number of alternations (the total number of arm entries minus 2) were calculated. The SAB score was the ratio of (the actual alternations/ the maximum number of alternations).

### Object-based attention test

The procedures for object-based attention test were adapted from the study previously described to fit the facilities [26]. Mice were placed in empty chambers (the exploration chamber and the test chamber, 33 × 33 ×  40 cm, L × W × H) each for 10 min on day 1–2 for habituation. On day 3 for the test phase, five objects (A, B, C, D, E) of the same size but different shapes (square-, star-, heart-, hexagon-, and cross-shaped) were placed in the chamber. Mice were allowed to explore freely in the chamber for 5 min during the exploration session and then they were transferred to a test chamber containing a familiar (square-shaped, object A) and a new (round-shaped, object F) object in less than 10 seconds and allowed to explore these two objects for a 5 min of retention session. Throughout the study, the position of each object in the exploration and test chambers was fixed. The recognition index (RI), calculated for each mouse, was expressed as the ratio of (TF × 100)/(TA + TF), where TA and TF are the time spent on object A and object F during the retention session, respectively. Another recognition index calculated in this test was expressed as the ratio of (TA × 100)/(TA + TD), where TA and TD are the time spent on object A and object D during the exploration session, respectively. Noldus software (EthoVision XT 15.0, Noldus Technology, Netherland) was used to monitor and track the movement of mice.

### Cliff avoidance reaction (CAR)

The cliff avoidance reaction (CAR) is used to evaluate maladaptive impulsive rodent behavior, especially for ADHD animal models [27]. The apparatus included a round wooden platform (20 cm in diameter and 2 cm in thickness) and a heavy iron rod (50 cm in height) below. The test began with placing mice on the platform with the forelimbs near the edge of the platform and lasted for 30 min. Falling from the platform once was recorded as one impaired CAR. After falling down, mice were placed back on the platform immediately. The distance traveled on the platform in the 30 min and the latency from the beginning until falling and the number of falls were recorded with Noldus software. The incidence of impaired CAR was calculated as a percentage index for each group, as follows: % CAR = (the number of mice that stay on the platform/the total number of mice) × 100.

#### Differential reinforcement of low rate schedule (DLR)

The procedures for DLR were adapted from the study previously described to fit the facilities [28, 29]. The apparatuses used were 4 operant chambers, each containing a touchscreen box (Campden Instruments, 89540 A). Briefly, the touchscreen box was equipped with 2 nose poke touchscreens, a loudspeaker, a liquid dispenser and a liquid tray with a LED light above it. Mice were weighed and handled for 1 min in the first 3 days and then underwent a food deprivation until their body weight reduced to 85–90% of their free-feeding weight. Strawberry milkshake was provided to the mice in their home cages for 2 days immediately before the training phase. The training phase can be divided into 3 stages under a continuous reinforcement schedule.

In the first stage, food-deprived mice were first trained to approach the tray after an LED light and an auditory stimulus to obtain a strawberry milkshake reward (10 μL). In the second stage, mice are trained to nosepoke the touchscreen, where an image pseudo-randomly appears on one of the two screens. Mice nosepoking the screen without an image received a strawberry milkshake reward (10 μL), while nosepoking the screen with an image resulted in a five times greater reward (50 μL). In the third stage, mice nosepoking the screen with an image received a strawberry milkshake reward (10 μL), while nosepoking the screen without an image received no reward. All the training stages lasted for at least 3 daily sessions of 30 min, or until a performance criterion (>30 rewarded responses within a session) was reached.

After the training phase, the DLR task was conducted. In this task mice were required to wait for a period of time (inter-response-time, IRT) between 2 consecutive nosepoke responses. Premature nosepoke reset the delay and resulted in no reward. Mice were trained and tested successively on DRL-4 s, DRL-6 s, DRL-8 s, DRL-10 s, DRL-15 s, each for 3 daily sessions of 30 min (30 trials within a session) and the result of the final session was analysed. The ratio between reinforcements and the total number of responses were calculated for DRL-4 s, DRL-6 s, DRL-8 s, DRL-10 s and DRL-15 s. Responses with IRT < 1 s were regarded as bursting response, while responses with IRT > 30 s were regarded as long IRT. The number of bursting responses and long IRT were calculated for DRL-15 s. For qualitative assessment of timing behavior, the relative frequency distributions of IRTs were generated, using a 5 s bin size.

#### Pre-pulse inhibition test (PPI)

Experiments were performed in sound attenuating test chambers (33 × 35 × 48 cm, L × W × H) equipped with a commercial startle reflex system in each chamber (SR-LAB Startle Response, San Diego Instruments, USA). First 5 min of the test is the acclimation phase with 50 dB acoustic background noise. Then 120 dB startle pulse (20 ms) was given to mice 12 times. In 48 subsequent experiments, the scare sound appeared either alone or after a random delay of 100 ms at three pre-pulse intensifies (65, 72, and 83 dB, 20 ms). The average interval between each trial was 30 s (random number from 20 to 40). The average startle amplitude during the 100 ms following the onset of each startle stimulus was recorded automatically. The first 12 trials are used to measure the baseline of acoustic startle response but excluded in the PPI analysis. The amount of PPI was expressed as the percentage decrease in the amplitude of the startle reactivity caused by presentation of the pre-pulse, which was calculated as follows: % PPI = 100 - {[(startle response for pre-pulse + pulse)/ (startle response for pulse alone)] × 100}.

### Drug administration

Methylphenidate (MPH) is a widely-used in the treatment of patients with ADHD [30]. MPH effects in a dose-dependent manner and high-dose of MPH can cause adverse effects, such as anxiety and aggressivity [31]. The dose of 2.5 mg/kg is reported to be suitable according to previous works [32]. To understand the mechanism underlying the effect of MPH treatment on short-term synaptic plasticity in Med23-deficient granule cells, the noncompetitive N-methyl-d-aspartate (NMDA) receptor antagonist dizocilpine-MK801(M107, Sigma, USA) was applied [33]. Mice received a single intraperitoneal injection of MK801 with the dose of 1 mg/kg 30 min before MPH treatment. Both MPH (0.1 mg/ml) and MK801 (0.1 mg/ml) were dissolved in saline and these compounds were prepared on the day of the experiments.

### In vitro patch-clamp recording

Hippocampal slices were prepared from mice by using procedures described previously [34]. The mouse brain was quickly removed and placed in ACSF bubbled with saturated gas (95% oxygen and 5% carbon dioxide) and cut into 350-μm-thick slices using a vibratome (Leica VT1000S, Leica Microsystems, Germany). All recording slices were carried out in the recording chamber, maintained at RT with standard ACSF, and placed on the Nikon microscope stage (600-FN, Nikon, Japan). The stimulation electrode was a pair of Teflon-coated 90 platinum/10% iridium wires. The recording electrode (1–3 mΩ) was pulled from borosilicate glass capillaries (1.5 mm outer diameter, 0.84 mm inner diameter, World Precision Instruments) with a Brown-Flaming micropipette puller (P-97; Sutter Instruments Company, USA), filled with ACSF. The fEPSP in the perforant path extending from layer II of the entorhinal cortex (EC) was recorded in the molecular layer of the DG area of the hippocampus by stimulation (0.1 ms duration). As a comparison, the fEPSP in the SC-CA1 pathway was recorded in the stratum radiatum of the dorsal CA1 area of the hippocampus by stimulation of the Schaffer collaterals (SCs) [35]. The GABA-dependent inhibitory transmission was not blocked during fEPSP recording. For input-output recordings, fEPSP slopes were recorded by increasing the intensity of stimulation (0.1 ms pulse width) from 0 with 20 µA increments. For paired-pulse ratio (PPR), the stimulation intensity was adjusted to give an fEPSP slope of 50% of the maximum, and a pair of stimuli with different intervals, including 50 ms, 100 ms, 150 ms, 200 ms, and 1000 ms was given successively for three times. The average value of three successive rounds was used for PPR analysis. The PPR was calculated as the ratio of the slope of the 2nd fEPSP to the slope of the 1st fEPSP. All data were recorded by using the pCLAMP 10 software (Molecular Devices). The signal collection was obtained by using Multiclamp 700B and Digidata 1440 A (Molecular Devices).

### Statistical analysis

Statistical analyses were performed by GraphPad Prism 9.5.1 software (GraphPad Prism, USA). All values were expressed as the mean ± SEM. The number of samples indicates biological replicates and also are indicated with scattered dots in the bars in the figures. For analysis of spine morphology, more than 3 mice were included in each group. For behavioral experiments, over 8 mice are included in each group. Comparisons were made using Student’s t-test, multiple t-test, one-way or two-way ANOVA with Bonferroni correction as mentioned in each figure legend. Statistical significance was set at p < 0.05.

### Study approval

Animal care practices and all experiments were reviewed and approved by the Animal Committee of Laboratory Animal Center, Fudan University, Shanghai, China (202408001Z).

## Results

### Med23 deletion leads to severe hypoplasia of DG

**Fig. 1:**
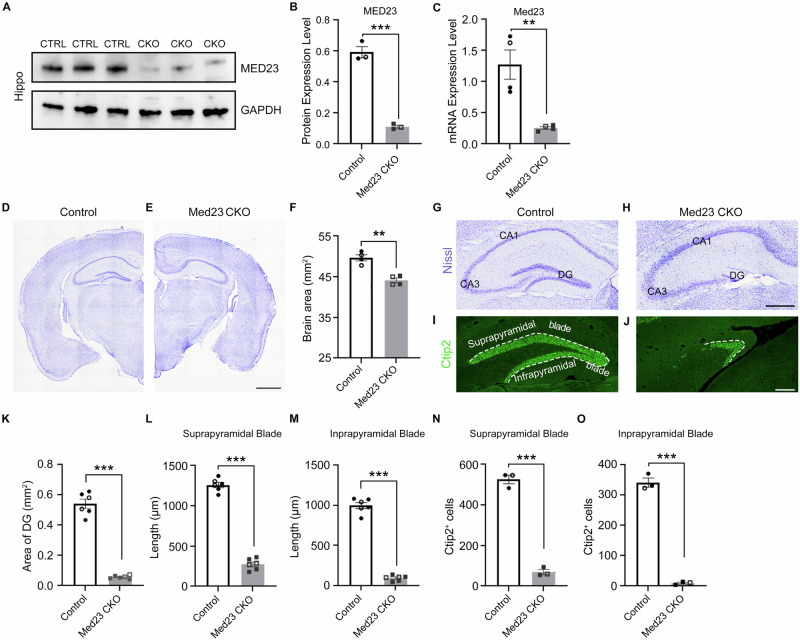
Med23 deficiency results in abnormal morphogenesis of dentate gyrus. **A** Immunoblot of Med23 using hippocampal tissues from Med23 CKO mice and control mice. Med23 protein expression level quantified by western blot (**B**: t = 12.82, df=4, *P* = 0.0002) and mRNA expression level measured by RT-qPCR (**C**: t = 4.331, df=6, *P* = 0.0049) in adult hippocampus of Med23 CKO mice and control mice. Med23 was successfully deleted in hippocampi in Med23 CKO mice. N = 3–4 mice for each group. Error bars in graphs represent mean ± SEM. **P < 0.01, ***P < 0.001, two-tailed Student’s t test. **D**–**F** Nissl staining of coronal sections of the adult brains of Med23 CKO mice and control mice. The brain area of Med23 CKO mice was smaller than control mice (**F**: t = 5.872, df=6, *P* = 0.0011). Scale bar, 1000 µm. N = 4 mice for each group. Error bars in graphs represent mean ± SEM. **P < 0.01, two-tailed Student’s *t* test. **G**, **H** Nissl staining of adult Med23 CKO hippocampus and control showed significant malformation in the dentate gyrus without Med23. Scale bar, 500 µm. **I**, **J** Immunostaining granule cell marker of Ctip2. Scale bar, 100 µm. The size of DG (**K**: t = 15.90, df=10, *P* < 0.0001), the length of the suprapyramidal blades (**L**: t = 22.60, df=10, *P* < 0.0001) and infrapyramidal blades (**M**: t = 22.60, df=10, *P* < 0.0001), and the number of Ctip2^+^ granule cells within the suprapyramidal blades (**N**: t = 18.59, df=4, *P* < 0.0001) and infrapyramidal blades (**O**: t = 21.93, df=4, *P* < 0.0001) in Med23 CKO mice were significantly reduced compared to that of control. N = 3 or 6 for each group. Error bars in graphs represent means ± SEM. ***P < 0.001, two-tailed Student’s t test. Solid symbols represent male mice, and hollow symbols represent female mice. CA Cornu Ammonis, DG dentate Gyrus, Hippo hippocampus.

Previous study has reported that an allele mutation termed *snouty* in the Med23 can cause embryonical lethality in mice at embryonic day 10.5 [36]. To further investigate the role of Med23 in the brain development and brain functions in adult stage, we used Emx1-Cre mice to conditionally knock out Med23 in the cells produced in the Emx1-expressing cell lineage like the excitatory neurons and glia (but not in GABAergic neurons) of the cerebral cortex and hippocampus [37], and Med23 CKO mice survived to adulthood without detectable gross abnormality. The deletion of Med23 was confirmed by Western Blot and RT-qPCR in Med23 CKO mice (Fig. 1A–C). We used Nissl staining to examine the internal structure of adult brains, and found that the brain size was slightly reduced in Med23 CKO mice compared with control brain (Fig. 1D–F). Surprisingly, the DG region was almost invisible whereas the CA1-3 regions of the hippocampus remained intact in Med23 CKO mice relative to controls regardless of sex (Fig. 1G, H). To further quantify the cellular changes, Ctip2 immunostaining was used to mark granule cells in the DG, and it showed that the suprapyramidal and infrapyramidal blades were evident in control mice but they were much reduced in Med23 CKO mice, shown by significant reductions of Ctip2-positive area, a massive loss of Ctip2-positive cells and shortened length of Ctip2-labled blades compared with controls (Fig. 1I–O). These results indicate that the deletion of Med23 with Emx1-driven Cre affects brain development particularly the DG morphogenesis.

### Malformation of dendrite tree in DG of Med23 CKO mice

**Fig. 2:**
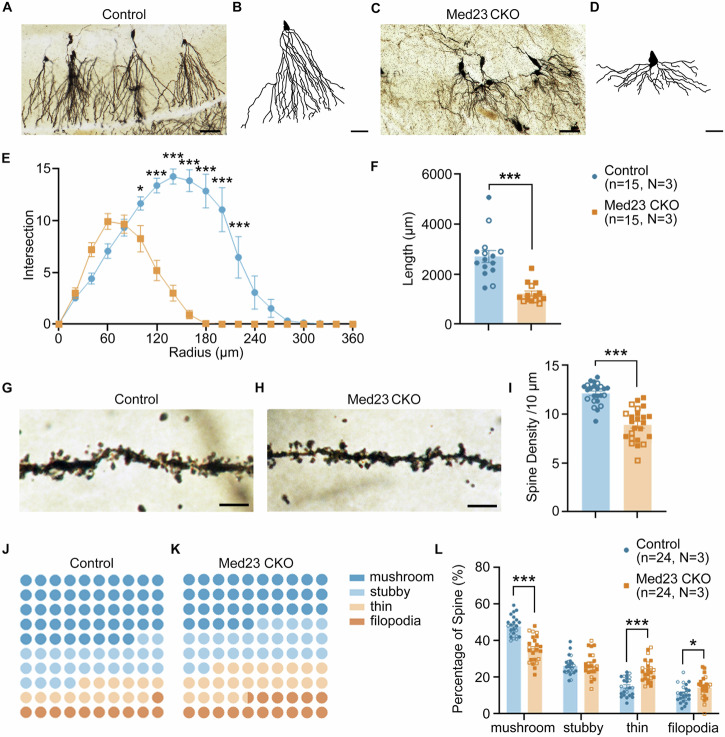
The morphology of dendrites and spines of DG granule cells are impaired after the ablation of Med23. Golgi staining of DG granule cells in suprapyramidal and infrapyramidal blades in control (**A**) and Med23 CKO mice (**C**). Scale bar, 100 µm. Examples of the reconstructed dendritic tree of DG granule cells from Med23 CKO (**B**) and control (**D**). Scale bar, 50 µm. The dendrites arranged in a chaotic manner in dentate gyrus after ablating Med23. **E** The cumulative length of dendritic arbor of granule cells was shorter in Med23 CKO mice than control (t = 5.723, df=28, *P* < 0.0001). n = 15 granule cells from N = 3 mice in control group. n = 15 granule cells from N = 3 mice in Med23 CKO group. Error bars in graphs represent means ± SEM. ***P < 0.001, two-tailed Student’s t test. **F** Sholl analysis for Control and Med23 CKO groups of granule cells (F_Radius_(18, 532) = 47.57, *P* < 0.0001; F_genotype_(1, 532) = 175.3, *P* < 0.0001; F_intersection_(18, 532) = 22.34, *P* < 0.0001;*P*_*100 μm*_ = 0.0484; *P*_*120 μm*_ < 0.0001; *P*_*140 μm*_ < 0. 0001; *P*_*160 μm*_ < 0. 0001; *P*_*200 μm*_ < 0. 0001; *P*_*220 μm*_ < 0. 0001). Error bars in graphs represent _*m*_eans ± SEM. *P < 0.05, ***P < 0.001, Two-way ANOVA with Bonferroni correction analysis. **G**, **H** Representative images of spines from secondary dendrites in DG granule cells in control and Med23 CKO mice. Scale bars, 5 μm. **I** Spine density per 10 µm of secondary dendrites in granule cells was decreased in Med23 CKO mice (t = 7.930, df=46, *P* < 0.0001). n = 24 dendrites from N = 3 mice in control group. n = 24 dendrites from N = 3 mice in Med23 CKO group. Error bars in graphs represent means ± SEM. ***P < 0.001, two-tailed Student’s t test. **J**, **K** dot plot graphs for the proportion of different categories of spines in control and Med23 CKO mice. Blue dots stand for mature spines with mushroom shape (dark-blue) and stubby shape (light-blue) and brown dots indicate immature spines which consist of thin-shaped (dark-brown) and filopodia-shaped (light-brown). The maturity of spines in Med23 CKO DG granule cells declined. **L** Percentage of mushroom-, stubby-, thin-, filopodia-shaped spines in control and Med23 CKO mice. n = 24 dendrites from N = 3 mice in control group. Med23 CKO mice exhibited reduced mushroom-shaped spines (t = 6.861, df=46, *P* < 0.0001) and increased thin- (t = 5.422, df=46, *P* < 0.0001), and filopodia-shaped (t = 2.071, df=46, *P* = 0.03) spines. n = 24 dendrites from N = 3 mice in Med23 CKO group. Error bars in graphs represent means ± SEM. **P < 0.01, ***P < 0.001, multiple and two-tailed Student’s t test. Solid symbols represent male mice, and hollow symbols represent female mice.

Since the presence of severe DG hypoplasia in Med23 CKO mice, we wondered how the morphology of residue granule cells was altered. We performed Golgi staining to visualize the dendritic architecture of granule cells. In control mice, the dendrites of granule cells were arranged orderly directing tangentially along the blade (Fig. 2A, B). Unlike control mice, the dendrites of granule cells were disorganized with overall shortened length, decreased complexity and reduced arborization in Med23 CKO mice (Fig. 2C, D). Then we analyzed the dendritic arbor of granule cells. Our results showed that Med23 deletion decreased the cumulative length of the dendritic arbor (Fig. 2E). Furthermore, we observed the complexity of dendritic arbors was significantly decreased in Med23 CKO compared with that in control mice (Fig. 2F).

Next, we analyzed the difference in the spine density of secondary dendrites in the DG granule cells between the two groups and found that dendritic spine density was significantly decreased in Med23 CKO mice compared with controls (Fig. 2G–I). The shape of spines is an important indicator reflecting neuronal function, and there are 4 categories based on spine shapes. Among them, mushroom- and stubby-shaped spines are considered as matured spines while thin- and filopodia-shaped spines represent the immature stage [38]. There was a remarkable reduction in the percentage of mushroom-shaped spines, while an increase in thin- and filopodia-shaped spines with no change in stubby-shaped spine in the granule cells of Med23 CKO mice compared with controls (Fig. 2J–L). Thus, the dendrites and spines of DG granule cells are severely affected in the absence of Med23.

### Med23 CKO mice exhibit ADHD-like behaviors

**Fig. 3:**
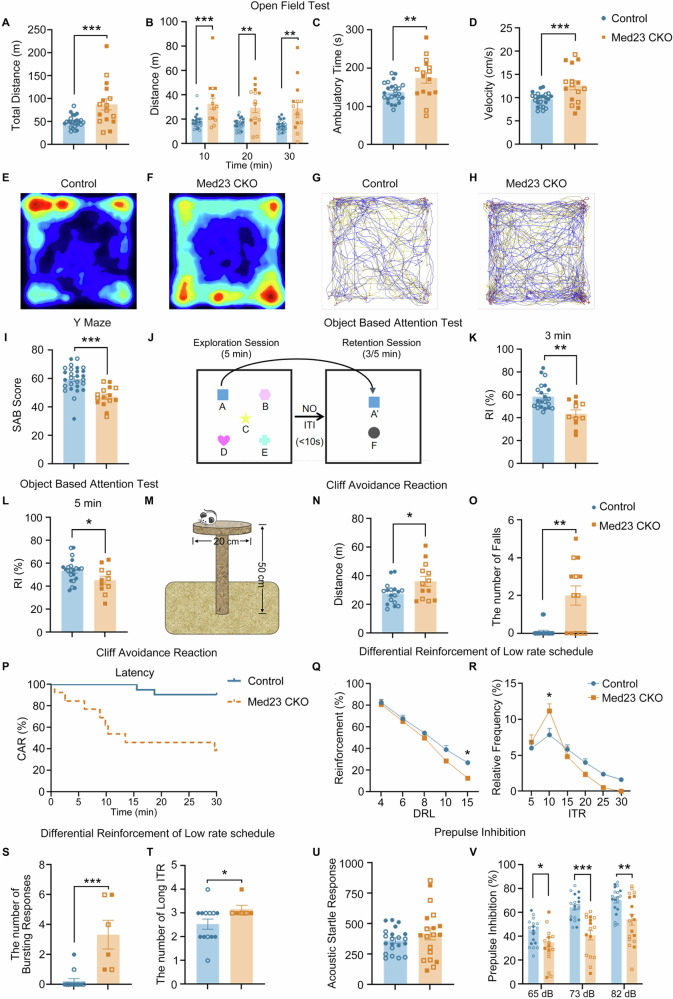
Med23 CKO mice exhibit ADHD-like behavioral phenotypes. The distance traveled in 30 min (**A**: t = 3.610, df=38, *P* = 0.0009), the distance traveled in per 10 min (**B**: F_time_(2, 114) = 0.9807, *P*_*time*_ = 0.3782; F_genotype_(1, 114) = 40.01, *P* < 0.0001; F_intersection_(2, 114) = 0.02397, *P* = 0.9763; *P*_10 min_ = 0.0007; *P*_*2*0 min_ = 0.00_*2*_0; *P*_*3*0 min_ = 0.0012), the ambulatory time (**C**: t = 2.920, df**=**38, *P* = 0.0059), and the average velocity in the ambulatory time (**D**: t = 3.410, df**=**38, *P* = 0.0016) revealed the hyperactivity of Med23 CKO mice in the open field test. N = 24 in control group, N = 16 in Med23 CKO group. Error bars in graphs represent means ± SEM. **p < 0.01, ***P < 0.001, two-tailed Student’s t-test for A and C, Two-way ANOVA with Bonferroni correction analysis for (**B**). **E–H** Representative heat map of mouse movement and movement track in open field test. **I** The SAB score of Y maze was lower in Med23 CKO mice (t = 4.501, df=39, *P* < 0.0001). N = 26 in control group, N = 15 in Med23 CKO group. Error bars in graphs represent means ± SEM. ***P < 0.001, two-tailed Student’s t test. **J** Schematic representation of the experiment methods of object-based attention test. The reduced recognition index for new object and familiar object in the 3-min retention session (**K**: t = 3.623, df=31, *P* = 0.0010) and 5-min retention session (**L**: t = 2.134, df=31, *P* = 0.0409) in Med23 CKO mice. N = 22 in control group, N = 11 in Med23 CKO group. Error bars in graphs represent means ± SEM. ***P < 0.001, two-tailed Student’s t test. **M** Schematic representation of the experiment apparatus of cliff avoidance reaction test. The distance traveled during the CAR test (**N**: t = 2.289, df=28, *P* = 0.0298), the number of falls (**O**: t = 3.730, df=12.40, *P* = 0.0027) and the time course of incidence of control and Med23 CKO mice (**P**: Chi square=12.58, df=1, *P* = 0.0004). Med23 CKO mice exhibited impulsive behaviors in CAR. N = 21 in control group, N = 13 in Med23 CKO group. Error bars in graphs represent means ± SEM. ***P < 0.001, two-tailed Student’s t-test for N, two-tailed Student’s t test with Welch correction analysis for O, Log-rank (Mantel-Cox) test for (**P**). **Q** The ratio between reinforcements and the total number of responses for the final session of DRL-4s, DRL-6s, DRL-8s, DRL-10s and DRL-15s in control and Med23 CKO group. F_genotype_(1, 85) = 10.80, *P* = 0.0015; F_DRL_(4, 85) = 115.1, *P* < 0.0001; F_intersection_(4, 85) = 1.369, *P* = 0.2515. *P*_DRL-4s_ > 0.9999; *P*_DRL-6s_ > 0.9999; *P*_DRL-8s_ > 0.9999; *P*_DRL-10s_ = 0.1314; *P*_DRL-15s_ = 0.0128. Error bars in graphs represent mean ± SEM. *P < 0.05, two-way ANOVA with Bonferroni correction analysis. **R** Mean relative frequency of IRT (inter-response-time: bin size=5 s) for the final session of DRL-15s in control and Med23 CKO group. Gray dotted lines represent 15-s IRT. F_genotype_(1, 102) = 0.5583, *P* = 0.4566; F_IRT_(5, 102) = 35.39, *P* < 0.0001; F_intersection_(5, 102) = 3.429, *P* = 0.0066. *P*_5s_ > 0.9999; *P*_10s_ = 0.0203; *P*_15s_ > 0.9999; *P*_20s_ = 0.8110; *P*_25s_ = 0.5498; *P*_30s_ = 0.8848. Error bars in graphs represent mean ± SEM. *P < 0.05, two-way ANOVA with Bonferroni correction analysis. **S** The number of bursting responses for the final session of DRL-15s in control and Med23 CKO group. t = 4.608, df=17, *P* = 0.0003. Error bars in graphs represent mean ± SEM. Two- tailed Student’s t test. **T** The number of long IRT for the final session of DRL-15s in control and Med23 CKO group. t = 2.307, df=16.48, *P* = 0.0343. Error bars in graphs represent mean ± SEM. Two- tailed Student’s t test with Welch correction analysis. N = 13 in control group, N = 6 in Med23 CKO group. **U** The baseline of acoustic startle response in the first 12 trials in PPI. t = 1.041, df=38, *P* = 0.3046. Error bars in graphs represent means ± SEM. Two-tailed Student’s t test. **V** The values of prepulse inhibiton test at 3 levels of prepulse intensities of 65 (t = 2.274, df=38, *P* = 0.0097), 73 (t = 5.395, df=38, *P* < 0.0001), and 82 (t = 3.260, df=38, *P* = 0.0012) dB in control and Med23 CKO mice. Med23 CKO mice showed defective sensorigating. N = 21 in control group, N = 19 in Med23 CKO group. Error bars in graphs represent means ± SEM. *P < 0.05, **P < 0.01, ***P < 0.001, multiple and two-tailed Student’s t-test. Solid symbols represent male mice, and hollow symbols represent female mice.

Considering the genetic association of Med23 mutation with neurodevelopmental diseases in human [19, 39] and the severe DG hypoplasia in adult Med23 CKO mice, we were encouraged to explore the behavioral alterations in the CKO mice. Data from both sexes were pooled together for statistical analysis based on the results that no differences were detected between the males and females (See Supplementary Materials). The open field test is a classic behavioral test commonly used to study spontaneous locomotor activity and exploratory behavior. We found significantly higher levels of locomotion in Med23 CKO mice including the total distance traveled in 30 min, the distance traveled per 10 min, ambulatory time, and average velocity (Fig. 3A–H). However, the times stayed in the center and peripheral zones, and vertical and stereotypic activities were similar between control and Med23 CKO mice (See Supplementary Materials). Thus, the hyperactivity phenotype shown by the horizontal activity is present in Med23 CKO mice.

Considering the roles of DG in learning and memory [40], the Y maze test was performed. The spontaneous alternation behavior in the Y maze test requires short-term working memory [25] and attention [41]. Mice is allowed to explore three arms of the maze and tend to visit previously unvisited arms driven by an innate curiosity. The SAB score of the Y maze was lower in Med23 CKO mice compared to control (Fig. 3I) showing impaired working memory and inattention.

We next utilized the object-based attention tests to further confirm the inattention phenotype [26] in the CKO mice. Med23 CKO mice possessed intact capability of distinguishing different objects and locations in the novel object recognition test and object location test (See Supplementary Materials), but they showed reduced recognition index in the object-based attention test (Fig. 3J–L), which can be inferred as a result of inattention because the nature of mice is to switch focus on the new object present in the test chamber, especially the exploration of 5 min and the retention session of 3 or 5 min with a 10 s interval that was too short for Med23 CKO mice to allocate and sustain attention rationally and effectively in the presence of multiple different objects in the exploration session and to identify the familiar object quickly in the retention session [26]. The reduced recognition index reflected that Med23 CKO mice were incapable of controlling attention with sustainability. Another typical symptom of ADHD is impulsivity, which is widely assessed by the cliff avoidance reaction test in rodents [27]. Med23 CKO mice not only traveled more distance than control mice on the platform but also jumped off earlier and more times from the platform during the whole 30-min test (Fig. 3M–P). Besides, we found significant deficits of Med23 CKO mice in the differential reinforcement of low rate schedule (DRL) performance (Fig. 3Q, R) reflecting possible defects in behavioral inhibition, executive control, short-term working memory and attention [28, 29]. Impulsivity and inattention shown by the increased number of bursting responses and long inter-response-time (ITR) was also observed in Med23 CKO mice (Fig. 3S, T).

Impaired sensorimotor gating is believed to be one of the key mechanisms underlying the behavioral abnormalities in ADHD [3], and the DG is a vital brain region in the sensorimotor gating process [42]. We thus performed the PPI test in the CKO mice. There was no difference in acoustic startle response which reflected the baseline startle-ness of the mice between the two groups (Fig. 3U). As expected, the CKO mice showed significant reductions in PPI scores at all levels of 65 dB, 73 dB and 82 dB compared with control mice (Fig. 3V).

We also detected depression-like behaviors in Med23 CKO mice, which associated with DG strongly [43] because depressive symptoms are common in ADHD patients [44]. We applied a series of behavioral tests for depression but found no difference between Med23 CKO mice and control mice of both sexes in the sucrose preference test, forced swimming test or tail suspension test (See Supplementary Materials). Taken together, Med23 CKO mice exhibit typical ADHD-like behaviors characterized by the hyperactivity, inattention, impulsivity and impaired working memory and sensorimotor gating.

### Methylphenidate (MPH) alleviates ADHD-like behaviors

**Fig. 4:**
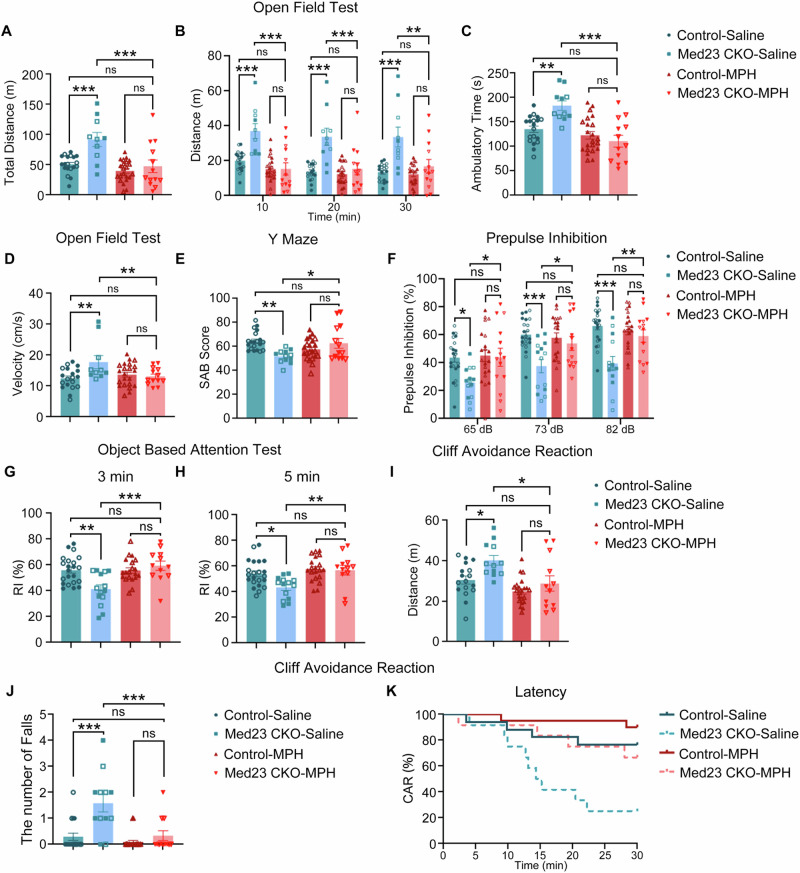
MPH alleviates ADHD-like behaviors and the deficits in the spines of granule cells in Med23 CKO mice. The distance traveled in 30 min (**A**: F_genotype_(1, 60) = 13.11, *P* = 0.0006; F_treatment_(1, 60) = 16.75, *P* = 0.0001; F_intersection_(1, 60) = 6.125, *P* = 0.0162; *P*_*CTRL-SAL-CKO-SAL*_ = 0.0007; *P*_*CTRL-SAL-CKO-MPH*_ = 0.9859; *P*_*CKO-SAL-CKO-MPH*_ = 0.0007; *P*_*CTRL-MPH-CKO-MPH*_ = 0.8260), the distance traveled in per 10 min (**B**: F_genotype_(1, 180) = 54.29, *P* < 0.0001; F_treatment_(1, 180) = 53.08, *P* < 0.0001; F_time_(2, 180) = 1.840, *P* = 0.1617; F_time×treatment_(2, 180) = 0.7821, *P* = 0.4590; F_time×genotype_(2, 180) = 0.6458, *P* = 0.5255; F_treatment×genotype_(1, 180) = 31.49, *P* < 0.0001; F_time×treatment×treatment_ (2, 180) = 0.0218, *P* = 0.9784; *P*_*10 min-CTRL-SAL-CKO-SAL*_ = 0.0008; *P*_*10 min-CTRL-SAL-CKO-MPH*_ = 0.9705; *P*_*10 min-CKO-SAL-CKO-MPH*_ < 0.0001; *P*_*10 min-CTRL-MPH-CKO-MPH*_ > 0.9999; *P*_*20 min-CTRL-SAL-CKO-SAL*_ < 0.0001; *P*_*20 min-CTRL-SAL-CKO-MPH*_ > 0.9999; *P*_*20 min-CKO-SAL-CKO-MPH*_ = 0.0007, *P*_*20 min-CTRL-MPH-CKO-MPH*_ = 0.9990; *P*_*30 min-CTRL-SAL-CKO-SAL*_ < 0.0001, *P*_*30 min-CTRL-SAL-CKO-MPH*_ = 0.9978, *P*_*30 min-CKO-SAL-CKO-MPH*_ = 0.0034, *P*_*30 min-CTRL-MPH-CKO-MPH*_ = 0.9701), ambulatory time (**C**: F_intersection_(1, 61) = 11.17, *P* = 0.0014; F_genotype_(1, 60) = 4.027, *P* = 0.0492; F_treatment_ (1, 61) = 22.43, *P* < 0.0001; *P*_*CTRL-SAL-CKO-SAL*_ = 0.0034; *P*_*CTRL-SAL-CKO-MPH*_ = 0.3310; *P*_*CKO-SAL-CKO-MPH*_ < 0.0001; *P*_*CTRL-MPH-CKO-MPH*_ > 0.9999), and average velocity in the ambulatory time (**D**: F_genotype_(1, 60) = 4.020, *P* = 0.0495; F_treatment_(1, 60) = 4.378, *P* = 0.0407; F_intersection_(1, 60) = 10.94, *P* = 0.0016; *P*_*CTRL-SAL-CKO-SAL*_ = 0.0043; *P*_*CTRL-SAL-CKO-MPH*_ > 0.9999; *P*_*CKO-SAL-CKO-MPH*_ = 0.0080; *P*_*CTRL-MPH-CKO-MPH*_ > 0.9999) were rescued by acute MPH administration in control and Med23 CKO mice in the open field test. N = 19 in Control-Saline group, N = 10 in Med23 CKO-Saline group, N = 22 in Control-MPH group, N = 13 in Med23 CKO-MPH group. Error bars in graphs represent means ± SEM. **P < 0.01, ***P < 0.001, Two-way ANOVA with Bonferroni correction analysis for (**A**, **C** and **D**), Three-way ANONA with Tukey correction analysis for (**B**). **E** The SAB score of Y maze. N = 20 in Control-Saline group, N = 12 in Med23 CKO-Saline group, N = 22 in Control-MPH group, N = 13 in Med23 CKO-MPH group. F_genotype_(1, 63) = 2.597, *P* = 0.1120; F_treatment_(1, 63) = 0.738, *P* = 0.3936; F_intersection_(1, 63) = 13.86, *P* = 0.0004; *P*_*CTRL-SAL-CKO-SAL*_ = 0.0028; *P*_*CTRL-SAL-CKO-MPH*_ > 0.9999; *P*_*CKO-SAL-CKO-MPH*_ = 0.0314; *P*_*CTRL-MPH-CKO-MPH*_ = 0.7929. Error bars in graphs represent means ± SEM. *P < 0.05, **P < 0.01, Two-way ANOVA with Bonferroni correction analysis. **F** The values of prepulse inhibiton test at 3 levels of prepulse intensities of 65 (F_genotype_(1, 67) = 4.907, *P* = 0.0302; F_treatment_(1, 67) = 5.287, *P* = 0.0246; F_intersection_(1, 67) = 3.804, *P* = 0.0553; *P*_*CTRL-SAL-CKO-SAL*_ = 0.0256; *P*_*CTRL-SAL-CKO-MPH*_ > 0.9999; *P*_*CKO-SAL-CKO-MPH*_ = 0.0484; *P*_*CTRL-MPH-CKO-MPH*_ > 0.9999), 73 (F_genotype_(1, 67) = 11.72, *P* = 0.0011; F_treatment_(1, 67) = 3.273, *P* = 0.0749; F_intersection_(1, 67) = 5.720, *P* = 0.0196; *P*_*CTRL-SAL-CKO-SAL*_ = 0.0006; *P*_*CTRL-SAL-CKO-MPH*_ > 0.9999; *P*_*CKO-SAL-CKO-MPH*_ = 0.0427; *P*_*CTRL-MPH-CKO-MPH*_ > 0.9999), and 82 dB (F_genotype_(1, 67) = 17.19, *P* < 0.0001; F_treatment_(1, 67) = 4.736, *P* = 0.0331; F_intersection_(1, 67) = 9.569, *P* = 0.0029; *P*_*CTRL-SAL-CKO-SAL*_ < 0.0001; *P*_*CTRL-SAL-CKO-MPH*_ = 0.9984; *P*_*CKO-SAL-CKO-MPH*_ = 0.0071; *P*_*CTRL-MPH-CKO-MPH*_ > 0.9999) in control and Med23 CKO mice. N = 22 in Control-Saline group, N = 14 in Med23 CKO-Saline group, N = 21 in Control-MPH group, N = 14 in Med23 CKO-M*P*H group. Error bars in graphs represent means ± SEM. *P < 0.05, **P < 0.01, ***P < 0.001, Two-way ANOVA with Bonferroni correction analysis. The recognition index for new object and familiar object of control and Med23 CKO mice in a 3-min retention session (**G**: F_genotype_(1, 64) = 3.904, *P* = 0.0492; F_treatment_(1, 64) = 10.24, *P* = 0.0021; F_intersection_(1, 64) = 10.98, *P* = 0.0015; *P*_*CTRL-SAL-CKO-SAL*_ = 0.0015; *P*_*CTRL-SAL-CKO-MPH*_ > 0.9999; *P*_*CKO-SAL-CKO-MPH*_ = 0.0006; *P*_*CTRL-MPH-CKO-MPH*_ > 0.9999) or 5-min retention session (**H**: F_genotype_(1, 64) = 4.880, *P* = 0.0308; F_treatment_(1, 64) = 10.73, *P* = 0.0017; F_intersection_(1, 64) = 3.679, *P* = 0.0596; *P*_*CTRL-SAL-CKO-SAL*_ = 0.0218; *P*_*CTRL-SAL-CKO-MPH*_ > 0.9999; *P*_*CKO-SAL-CKO-MPH*_ = 0.0094; *P*_*CTRL-MPH-CKO-MPH*_ > 0.9999). N = 22 in Control-Saline group, N = 14 in Med23 CKO-Saline group, N = 20 in Control-MPH group, N = 12 in Med23 CKO-MPH group. Error bars in graphs represent means ± SEM. *P < 0.05, **P < 0.01, ***P < 0.001, Two-way ANOVA with Bonferroni correction analysis. The distance traveled during the CAR test (**I**: F_genotype_(1, 59) = 8.811, *P* = 0.0043; F_treatment_(1, 59) = 13.73, *P* = 0.0005; F_intersection_(1, 59) = 1.568, *P* = 0.2155; *P*_*CTRL-SAL-CKO-SAL*_ = 0.0303; *P*_*CTRL-SAL-CKO-MPH*_ > 0.9999; *P*_*CKO-SAL-CKO-MPH*_ = 0.0149; *P*_*CTRL-MPH-CKO-MPH*_ > 0.9999), the number of falls (**J**: F_genotype_(1, 59) = 18.97, *P* < 0.0001; F_treatment_(1, 59) = 17.08, *P* = 0.0001; F_intersection_(1, 59) = 8.860, *P* = 0.0042; *P*_*CTRL-SAL-CKO-SAL*_ < 0.0001; *P*_*CTRL-SAL-CKO-MPH*_ > 0.9999; *P*_*CKO-SAL-CKO-MPH*_ = 0.0002; *P*_*CTRL-MPH-CKO-MPH*_ > 0.9999), and the time course of incidence (**K**: Chi square=9.08, df=3, *P* = 0.0003) of control and Med23 CKO mice. N = 17 in Control-Saline group, N = 12 in Med23 CKO-Saline group, N = 22 in Control-MPH group, N = 12 in Med23 CKO-MPH group. Error bars in graphs represent means ± SEM. *P < 0.05, ***P < 0.001, Two-way ANOVA with Bonferroni correction analysis for (**I**, **J**), Log-rank (Mantel-Cox) test for K. Solid symbols represent male mice, and hollow symbols represent female mice. CTRL-SAL control-saline group, CTRL-MPH control-MPH group, CKO-SAL Med23 CKO-Saline group, CKO-MPH Med23 CKO-MPH group.

MPH is the first-line medication to treat ADHD in clinics, and it is also widely used to evaluate ADHD-like behaviors in animal models [45]. We next explored whether MPH could mitigate these behavioral alterations in Med23 CKO mice. MPH is a short-acting stimulant, and the mice received acute MPH administration 30 min ahead of behavioral tests. In the open field test, MPH administration successfully rescued the hyperactivity in Med23 CKO mice with decreased total traveled distance, ambulatory time, velocity and distance traveled per 10 min (Fig. 4A–D). In addition, MPH also restored the ability of working memory shown by increased SAB scores in the Y maze (Fig. 4E) and improved sensorimotor gating shown by the data from the PPI (Fig. 4F). As to the impulsive and inattentional phenotypes, MPH administration was efficient to improve the recognition index in the object-based attention tests (Fig. 4G, H) and the performance in the cliff avoidance reaction test in Med23 CKO mice (Fig. 4I–K). These findings demonstrated that MPH treatment ameliorates ADHD-like behaviors in Med23 CKO mice.

### MPH rescues DG spinogenesis in Med23 CKO mice

ADHD is a developmental disorder that persists through childhood, adolescence and adulthood. MPH is often prescribed for a long time to achieve long-term pharmacological effects [46]. Since we found the deficits of spines in Med23-deficient granule cells and previous studies have shown that MPH is able to induce the spine formation [47], we thus treated the CKO mice with MPH administration for 2 weeks to investigate possible effect of MPH on dendritic spines. We found that the spine density of DG granule cells was significantly elevated in Med23 CKO mice after MPH treatment compared to Med23 CKO mice treated with saline. More importantly, the constitutions of 4 spine categories in MPH-treated Med23 CKO mice were fully recovered to the levels of control mice, revealing the chronic MPH administration restored the deficits in spine formation caused by Med23 deletion. Taken together, chronic MPH treatment enables the strengthening of neuroplasticity by enhancing the spinogenesis in the Med23-deficient granule cells (See Supplementary Materials).

### Impaired synaptic plasticity in DG of Med23 CKO mice

**Fig. 5:**
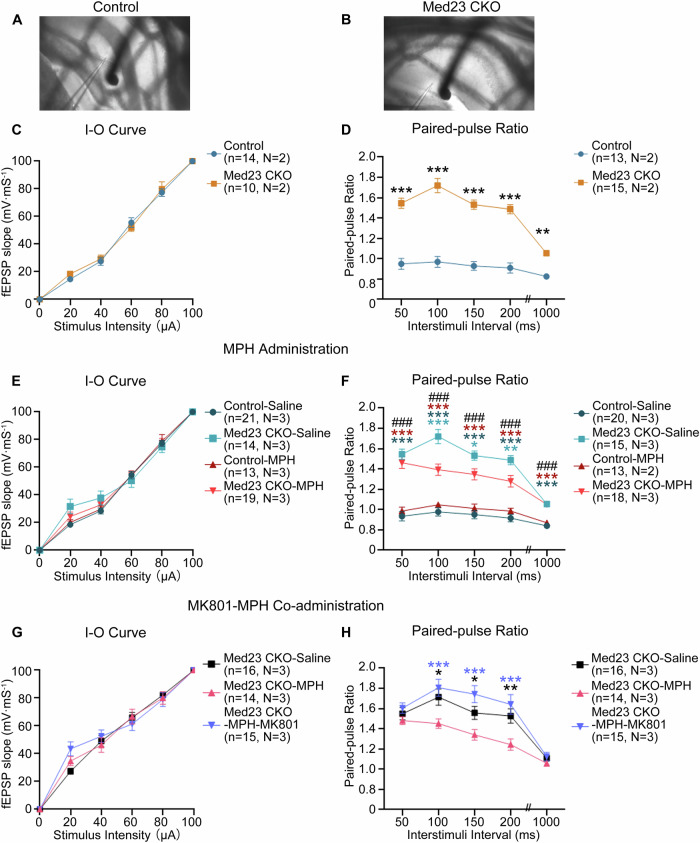
MPH facilitates the synaptic transmission via NMDA-receptors in Med23-deficient DG granule cells. **A**, **B** Representative images of the recorded areas in control and Med23 CKO mice. **C** The I-O curves for input-output recordings plotted with stimulus intensity against slope showed no difference in control and Med23 CKO mice. n = 14 from N = 2 mice in control group, n = 10 from N = 2 mice in Med23 CKO group. F_genotype_(1, 132) = 0.2424, *P* = 0.6233; F_intensity_(5, 132) = 462.5, *P* < 0.0001; F_intersection_(5, 132) = 0.5374, *P* = 0.7476. Error bars in graphs represent means ± SEM. Two-way ANOVA with Bonferroni correction analysis. **D** The PPR for short-term synaptic plasticity was higher in Med23 CKO mice than control mice. n = 13 from N = 2 mice in control group, n = 15 from N = 3 mice in Med23 CKO group. F_genotype_(1, 130) = 328.8, *P* < 0.0001; F_interval_(4, 130) = 19.82, *P* < 0.0001; F_intersection_(4, 130) = 8.161, *P* < 0.0001; *P*_50 ms_ < 0.0001; *P*_100 ms_ < 0.0001; *P*_150 ms_ < 0.0001; *P*_200 ms_ < 0.0001; *P*_1000 ms_ = 0.0055. Error bars in graphs represent means ± SEM. Two-way ANOVA with Bonferroni correction analysis. **E** The I-O curves in control and Med23 CKO mice treated with saline or acute MPH administration. n = 21 from N = 3 mice in control^-^saline group, n = 14 from N = 3 mice in Med23 CKO-saline group, n = 12 from N = 2 mice in control-MPH group, n = 19 from N = 3 mice in Med23 CKO-MPH group. At 20 μA: F_genotype_(1, 62) = 0.0856, *P* = 0.7708; F_treatment_(1, 62) = 3.987, *P*^=^0.0502; F_intersection_(1, 62) = 0.9318, *P* = 0.3381. At 40 μA: F_genotype_(1, 62) = 0.0121, *P* = 0.9128; F_treatment_(1, 62) = 2.708, *P* = 0.1049; F_intersection_(1, 62) = 0.1654, *P* = 0.6302. At 60 μA: F_genotype_(1, 62) = 1.220, *P* = 0.2737; F_treatment_(1, 62) = 1.533, *P* = 0.2204; F_intersection_(1, 62) = 1.288, *P* = 0.2607. At 80 μA: F_genotype_(1, 62) = 0.6601, *P* = 0.4196; F_treatment_(1, 62) = 0.6126, *P* = 0.4368; F_intersection_(1, 62) = 0.1313, *P* = 0.7184. Error bars in graphs represent means ± SEM. Two-way ANOVA with Bonferroni correction analysis. **F** The PPR with different interstimuli intervals in control and Med23 CKO mice treated with saline or acute MPH administration. n = 20 from N = 3 mice in control^-^saline group, n = 15 from N = 3 mice in Med23 CKO-saline group, n = 13 from N = 2 mice in control-MPH group, n = 18 from N = 3 mice in Med23 CKO-MPH group. At 50 ms: F_genotype_(1, 62) = 113.9, *P* < 0.0001; F_treatment_(1, 62) = 0.1050, *P*^=^0.7470; F_intersection_(1, 62) = 1.776, *P* = 0.1876; ### *P*_*CTRL-SAL-CKO-SAL*_ < 0.0001; ***(dark blue) *P*_*CTRL-SAL-CKO-MPH*_ < 0.0001; ***(dark red) *P*_*CTRL-MPH-CKO-MPH*_ < 0.0001. At 100 ms: F_genotype_(1, 62) = 106.4, *P* < 0.0001; F_treatment_(1, 62) = 5.9229, *P* = 0.0178; F_intersection_(1, 62) = 14.15, *P* = 0.0004; ### *P*_*CTRL-SAL-CKO-SAL*_ < 0.0001; ***(dark blue) *P*_*CTRL-SAL-CKO-MPH*_ < 0.0001; ***(sky blue) *P*_*CKO-SAL-CKO-MPH*_ = 0.0002; ***(dark red) *P*_*CTRL-MPH-CKO-MPH*_ = 0.0002. At 150 ms: F_genotype_(1, 62) = 92.60, *P* < 0.0001; F_treatment_(1, 62) = 1.699, *P* = 0.1973; F_intersection_(1, 62) = 6.645, *P* = 0.0123; ### *P*_*CTRL-SAL-CKO-SAL*_ < 0.0001; ***(dark blue) *P*_*CTRL-SAL-CKO-MPH*_ < 0.0001; *(sky blue) *P*_*CKO-SAL-CKO-MPH*_ = 0.0442; ***(dark red) *P*_*CTRL-MPH-CKO-MPH*_ < 0.0001. At 200 ms: F_genotype_(1, 62) = 91.21, *P* < 0.0001; F_treatment_(1, 62) = 2.379, *P* = 0.1281; F_intersection_(1, 62) = 9.532, *P* = 0.0030; ### *P*_*CTRL-SAL-CKO-SAL*_ < 0.0001; ***(dark blue) *P*_*CTRL-SAL-CKO-MPH*_ < 0.0001; **(sky blue) *P*_*CKO-SAL-CKO-MPH*_ = 0.0095; ***(dark red) *P*_*CTRL-MPH-CKO-MPH*_ = 0.0002. At 1000 ms: F_genotype_(1, 62) = 100.4, *P* < 0.0001; F_treatment_(1, 62) = 0.5138, *P* = 0.4762; F_intersection_(1, 62) = 0.690, *P* = 0.4094; ### *P*_*CTRL-SAL-CKO-SAL*_ < 0.0001; ***(dark blue) *P*_*CTRL-SAL-CKO-MPH*_ < 0.0001; ***(dark red) *P*_*CTRL-MPH-CKO-MPH*_ < 0.0001. Error bars in graphs represent means ± SEM. Two-way ANOVA with Bonferroni correction analysis. **G** The I-O curves of Med23 CKO mice treated with saline or acute MPH administration and Med23 CKO mice pretreated with MK801 before MPH administration. F_treatment_(2, 252) = 0.5328, *P* = 0.5876; F_intensity_(5, 252) = 314.6, *P* < 0.0001; F_intersection_(10, 252) = 1.375, *P* = 0.1919. Error bars in graphs represent means ± SEM. Two^-^way ANOVA with Bonferroni correction analysis. **H** The PPR with different interstimuli intervals in Med23 CKO mice treated with saline or acute MPH administration and Med23 CKO mice pretreated with MK801 before MPH administration. Pretreatment with MK801 (1 mg/kg) effectively blocked MPH-induced PPR improvement. F_treatment_(2, 215) = 22.01, *P* < 0.0001; F_interval_(4, 215) = 34.62, *P* < 0.0001; F_intersection_(8, 215) = 1.657, *P* = 0.1105. At 50 ms: *P*_*CKO-SAL-CKO-MPH*_ > 0.9999; *P*_*CKO-SAL-CKO-CO*_ > 0.9999; *P*_*CKO-MPH-CKO-CO*_ = 0.5383. At 100 ms: * (black) *P*_*CKO-SAL-CKO-MPH*_ = 0.0110; *P*_*CKO-SAL-CKO-CO*_ = 0.8621; ***(blueviolet) *P*_*CKO-MPH-CKO-CO*_ = 0.0004. At 150 ms: * (black) *P*_*CKO-SAL-CKO-MPH*_ = 0.0485; *P*_*CKO-SAL-CKO-CO*_ = 0.1071; ***(blueviolet) *P*_*CKO-MPH-CKO-CO*_ < 0.0001. At 200 ms: ** (black) *P*_*CKO-SAL-CKO-MPH*_ = 0.0053; *P*_*CKO-SAL-CKO-CO*_ = 0.5772; ***(blueviolet) *P*_*CKO-MPH-CKO-CO*_ < 0.0001. At 1000 ms: *P*_*CKO-SAL-CKO-MPH*_ > 0.9999; *P*_*CKO-SAL-CKO-CO*_ > 0.9999; *P*_*CKO-MPH-CKO-CO*_ > 0.9999. Error bars in graphs represent means ± SEM. Two-way ANOVA with Bonferroni correction analysis. CKO-SAL: Med23 CKO-Saline group; CKO-MPH: Med23 CKO-MPH group; CKO-CO: Med23 CKO-MPH-MK801 group.

The DG receives excitatory inputs from the entorhinal cortex (EC) and transmits processed information to CA3 subregion [8], which plays a vital role as the junction of the hippocampal trisynaptic circuit for the processing and integration of sensory information underlying learning and memory, as well as sensorimotor gating [42, 48]. Patients with ADHD often have impaired sensory processing in both adults and children [49, 50], and it is also present in our Med23 CKO mice revealed by the PPI test. We thus hypothesized that the DG malformation may lead to malfunction of the hippocampal circuit contributing to the ADHD-like behaviors in Med23 CKO mice. We utilized the patch-clamp recording on the hippocampal slices to evaluate the functional connectivity from the EC to DG. The evoked field excitatory postsynaptic potentials (fEPSPs) in the DG were recorded after electrical stimulation of the EC (Fig. 5A, B). When we stimulated EC only once, we detected no difference between control and Med23 CKO mice in the input-output (I-O) curves (Fig. 5C), which means that Med23 ablation did not affect the basal synaptic transmission from the EC to DG granule cells. When 2 consecutive stimuli were applied in the EC with a short interval, the 2nd fEPSP received in DG is supposed to be depressed by the 1st fEPSP with the paired-pulse ratio (PPR) less than the first one as shown in control mice. However, the PPR differed significantly with a remarkable increase for different interstimuli intervals from 50 ms to 1000 ms in Med23 CKO mice (Fig. 5D) implying dysfunctional short-term synaptic plasticity of DG granule cells. We also tested the connection between Schaffer collaterals (SCs) from CA3 and the stratum radiatum of the dorsal CA1 area, but found that PPR in this pathway was unchanged (See Supplementary Materials). The PPR is a reliable indicator for evaluating sensory gating ability [51], and the impaired sensory gating is involved in the pathogenesis of ADHD [3]. Based on these, it is very likely that the malfunction of EC-DG PPR contributes to ADHD-like behaviors in Med23 CKO mice.

We also performed the slice recording with bath application of MPH, and found that the deficits of PPR were significantly restored in Med23 CKO mice, although MPH could not correct the ratio back to the level of control mice (Fig. 5E, F). MPH impacts the glutamatergic homeostasis through NMDA receptors [52], and we therefore applied MK801, an antagonist for NMDAR in the slice from Med23 CKO mice. MK801 and MPH co-administration did not change the I-O curve reflecting the intact basic synaptic transmission from the EC to DG (Fig. 5G), but the partially-restored PPR by MPH was abolished by administration of MK801 in hippocampal slices from Med23 CKO mice, almost returning to the PPR level of Med23 CKO mice treated with saline (Fig. 5H). Thus, the effect of MPH-rescued DG synaptic plasticity in Med2 CKO mice is achieved at least partially by NMDAR-mediated mechanisms.

## Discussion

In this study, we demonstrated that Med23 CKO mice developed severe DG hypoplasia with the malformation of dendrites and spines in the granule cells and deficits in the short-term synaptic plasticity. Med23 CKO mice also showed ADHD-like behaviors, with the traits including the hyperactivity, inattention, impulsivity and impaired working memory and sensorimotor gating. In addition, the majority of these abnormalities in CKO mice were normalized by MPH, a first-line drug for ADHD treatment. Our findings suggest that DG malformation resulted from Med23 deficiency might linked with the etiology and pathology of ADHD.

Considering the similar phenotypes of ADHD patients and the usefulness of MPH administration in Med23 CKO mice, Med23 CKO mice may serve as a novel animal model for studying ADHD-like behaviors and its deficiency-related brain diseases. With regard to the face validity, Med23 CKO mice exhibit locomotor hyperactivity, attention deficit and impulsivity, all of which fit with ADHD [53]. Furthermore, Med23 CKO mice showed deficits in working memory and sensorimotor gating, conditions comorbid with ADHD [3, 54]. Considering the predictive validity, Med23 CKO mice responded to the administration of the classic drug MPH with alleviated ADHD-like behavioral changes, consistent with the therapeutic effects of MPH in ADHD patients [55]. Finally, the reduced volume and altered functional connectivity of the hippocampus were reported in patients with ADHD [6, 7, 56]. Besides, decreased Med23 RNA expression was found in the whole blood of ADHD patients, which also hints at the construct validity of Med23 CKO model [22]. Thus, we suggest that Med23 CKO mice might be a novel ADHD mouse model in terms of the construct, face and predictive validities. Nowadays, widely-used validated rodent models bear a phenotypic resemblance to ADHD in which they possess genetic and behavioral abnormalities found in ADHD patients including typical hyperactivity, inattention and impulsivity and drug reaction [57]. We put forward the first ADHD model linking Med23 deficiency, DG malformation and the etiology and pathology of ADHD together. Although considering the overlapping symptoms between ADHD and comorbid psychopathologies represented in the clinic [58], Med23 CKO mice as an ADHD animal model had its limitations because the interpretation of the behavioral consequences deriving from the genetic mutation may also be associated with other cognitive disorders like schizophrenia, learning disabilities or dementia [59].

Med23 has been reported to be related with intellectual disability, epilepsy and Alzheimer’s disease [20, 21, 60]. Although global development delay and microcephaly are common features for patients with Med23 mutations [19], the role of Med23 in brain development and other development-associated diseases was not well clarified. Our previous study reported that Med23 regulates adult hippocampal neurogenesis [23]. In this study, we found that embryonic Med23 deletion resulted in extremely severe hypoplasia of DG with disorganized dendrites, reduced and immature spines in the granule cells. Our work revealed a surprisingly close connection between Med23 and DG development for the first time. Further studies are needed to explore the molecular mechanism underlying Med23-deficiency-induced DG malformation and the appearance of ADHD-like behaviors.

The ADHD-like behaviors featured with hyperactivity, inattention, impulsivity and impaired sensorimotor gating and working memory in Med23 CKO mice whose DG has great developmental defects giving us a new piece of evidence for the potential role of DG in ADHD. The DG is less focused in the clinical studies, but the reduction of volume and altered functional connectivity with the hippocampus have been reported in patients with ADHD [6, 13, 56]. Med23 CKO mice exhibit impaired neural connection from the EC to DG shown by elevated PPR. The projection from the EC to DG (the perforant path) is important in learning, memory and sensorimotor gating^48^, the defects in corresponding abilities were observed in the ADHD-related behaviors of Med23 CKO mice reflected by low SAB score in Y maze and impaired PPI. Therefore, the malformation of DG in Med23 CKO mice hints that the dysfunction of DG in learning, memory and sensorimotor gating may contributes to the pathology of ADHD. Possibly, the altered DG-based neuronal connection may be an important factor in the pathogenesis of ADHD.

MPH exerts its pharmacological effects by inhibiting the dopamine transporter and the norepinephrine transporter preferentially, resulting in increased levels of neurotransmitters in the synapses [61]. In this study, we found the effects of MPH administration on the PPR were blocked through the blockade of NMDAR with MK801, which means MPH ameliorated the impaired PPR in a NMDAR-dependent manner. It was reported that MPH treatment is able to alter the protein levels of NMDAR subunits [62], which may be a possible explanation for our findings. Another possible hypothesis we propose for this finding is that MPH may facilitate NMDAR-mediated synaptic transmission. Previous studies demonstrate MPH regulates NMDAR-mediated excitatory synaptic currents (EPSC) through dopamine receptors or adrenergic receptors [63]. Another in vitro study suggests that the enhancement of NMDAR-mediated EPSC after MPH treatment is mediated by Sigma-1 receptors instead of adrenergic or dopamine receptors [64], and MPH may influence NMDAR activity via other receptors in Med23 CKO mice.

In this study, we focused on the relationship between the DG and ADHD-like behaviors, but did not address the potential contributions of the cortical and CA1 regions with the inactivation of Med23. Considering the forebrain developmental deficits existing in Med23 CKO mice and abnormalities in cortical structures and functions of patients with ADHD [65, 66], it is possible that the consequence of cortical deletion of Med23, along with DG developmental disorder, give rise to the behavioral phenotypes in Med23 CKO mice. Besides, the brain functions as an integrated system, and the pathogenesis of ADHD involves multiple brain regions. Although MPH can partially correct the PPR from EC to DG, the therapeutic effects of MPH with compensating for synaptic transmission as well as alleviating abnormal behaviors may also be achieved through influencing other structures related to ADHD symptoms, such as the striatum, basal ganglia, and cerebellum [67, 68]. Further studies are needed to expand our understanding of Med23-involved pathogenesis of ADHD.

In summary, our findings indicate that Med23 is a crucial factor of the morphogenesis of DG and the pathogenesis of ADHD, thus providing valuable information to advance the deciphering of the potential genetic and pathological underpinnings of ADHD.

## Supplementary information

supplemental materials

## Data Availability

All data supporting the findings are available within the article, supplemental materials and Supporting Data Values files.
