# Supplementary material for: Central Med23 deficiency leads to malformation of dentate gyrus and ADHD-like behaviors in mice
Source: Neuropsychopharmacology. 2025 Mar 20;50(8):1224–36. doi: 10.1038/s41386-025-02088-1 (PMC12170885; doi:10.1038/s41386-025-02088-1)
Supplement: Supplementary file 1 — supplemental materials [file 41386_2025_2088_MOESM1_ESM.docx]

**SUPPLEMENTAL MATERIALS**

**Methods**

**Novel object recognition/Object location recognition Test**

The novel object recognition test was conducted in a big soundproof box (40 × 40 × 40 cm, L × W × H). The test was performed as described previously[1]. On day 1-2 of habituation sessions, mice were put in the empty box for 20 min per day. On day 3 of test session, two identical objects were placed symmetrically in the box and mice were allowed to explore for 15 min. After 1 h, one familiar object was replaced by a novel one and mice were allowed to explore for another 15 min. The two kinds of objects used in the test have similar size and smell but different shape and texture. The time spent exploring each object was scored when the subject was sniffing towards the object within 2 cm. Recognition index (RI) was defined as [(time spent exploring the novel object – time spent exploring the familiar object)/time spent exploring the novel object and the familiar object] × 100%.

The novel object recognition test was performed as described previously with some modifications[2]. In the object location recognition test, all the steps were same as in novel object recognition test except replacing one familiar object with a novel object. This step was removing one of the familiar objects to a new location in the box. Recognition index (RI) was defined as [(time spent exploring the novel location – time spent exploring the familiar location)/time spent exploring the novel location and the familiar location] × 100%.

**Sucrose preference test**

Sucrose preference test was conducted as described previously[3]. All stages of the test were conducted at the same time of day. During the initial 24-hour training phase, each cage was equipped with two drinking tubes containing a 2% w/v sucrose solution. Then the next day, one bottle with 2% sugar solution and another bottle with regular water were provided to mice. Following the training phase, the mice underwent a 24-hour period of water and food deprivation. Subsequently, the mice were given the opportunity to freely choose between two bottles for 24 hours: one filled with the sucrose solution and the other with water. After the first 12 hours, the positions of the bottles in the cage were switched. The consumption of sucrose and water was recorded separately before and after the test. Sucrose preference was calculated as (sucrose intake/total intake) × 100%, where the total intake value is the sum of the water intake value and the sucrose intake value.

**Tail suspension test**

Tail suspension test was conducted as described previously[3]. For this test, mice were suspended 30 cm above the floor using an adhesive tape placed approximately 1 cm from the tail's end on a metal hook. Initially, most mice made efforts to escape from the suspended status. However, after a period of struggling, they entered a state of intermittent immobility, referred to as "behavioral despair." The duration of this state was recorded as the immobility time. A video camera was used to capture the mice's activities, and the immobility time during the final 5 minutes of a 6-minute testing period was measured.

**Forced swimming test**

Forced swimming test was conducted as described previously[3]. For this test, mice were placed in a transparent acrylic cylinder (15 cm in diameter and 30 cm in height) which was filled with purified tap water to a depth of 20 cm for 6 min individually. The first 1 min were spent for adaptation, and the following 4 min were analyzed. Immobility time was evaluated as floating or no active movements except those necessary for the mouse to keep its head above water.

**Supplemental Figures**


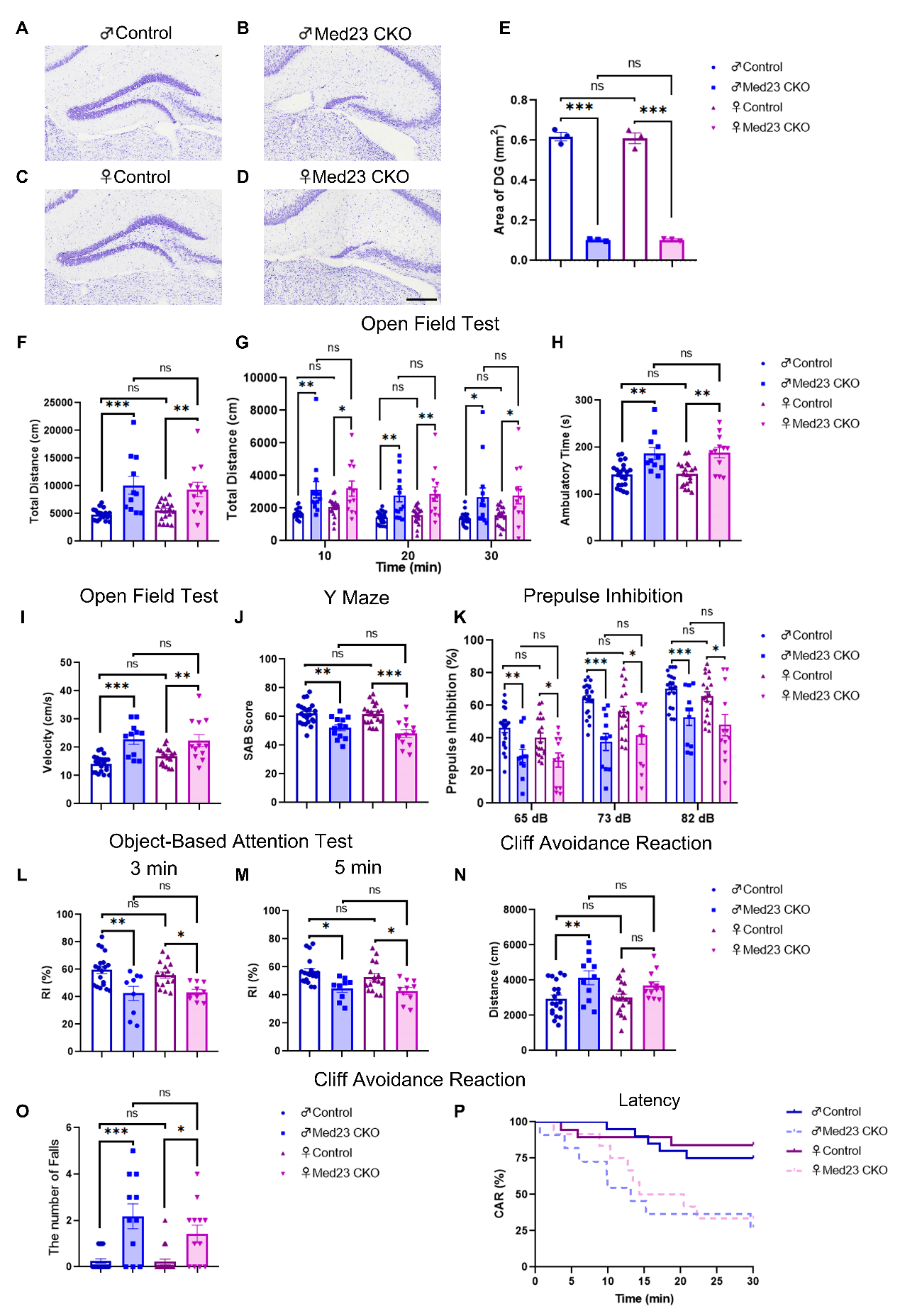


**Fig. S1: Both sexes exhibited ADHD-like behaviors after Med23 deletion. A-E** Both sexes of Med23 CKO mice showed severe atrophy of DG with obviously small area. F_genotype_(1, 8)=867.8, *P***<**0.0001; F_sex_(1, 8)=0.0598, *P*=0.8129; F_intersection_(1, 8)=0.0779, *P*=0.7872; *P_male-CTRL-K_***<**0.0001; *P_female CTRL-CKO_***<**0.0001; *P_male CTRL-female CTRL_*>0.9999; *P_male CKO-female CKO_*>0.9999. Error bars in graphs represent means ± SEM. ****P*<0.001, Two-way ANOVA with Bonferroni correction analysis for E. **F-I** Both sexes of Med23 CKO mice showed hyperactivity in the open field test with increased distance traveled in 30 min (**F**: F_genotype_(1, 59)=29.19, *P***<**0.0001; F_sex_(1, 59)=0.0561, *P*=0.8135; F_intersection_(1, 59)=0.6726, *P*=0.4154; *P_male-CTRL-K_*=0.0003; *P_female CTRL-CKO_*=0.0002; *P_male CTRL-female CTRL_*>0.9999; *P_male CKO-female CKO_*>0.9999), the distance traveled in per 10 min (**G**: F_genotype_(1, 177)=76.37, *P*<0.0001; F_sex_(1, 177)=0.1468, *P*=0.7021; F_time_(2, 177)=2.635, *P*=0.0745; F_time×sex_(2, 177)=0.0171, *P*=0.9831; F_sex×genotype_(1, 177)=1.760 *P*=0.1864; F_time×genotype_(2, 177)=0.0009, *P*=0.9991; F_time×sex×genotype_(2, 177)=0.0562, *P*=0.9453; *P_10 min_-_male-CTRL-CKO_* =0.0019; *P_10 min_-_female CTRL-CKO_*=0.0321; *P_10 min_-_male CTRL-female CTRL_*=0.9916; *P_10 min_-_male CKO-female CKO_*>0.9999; *P_20 min_-_male-CTRL-CKO_* =0.0058; *P_20 min_-_female CTRL-CKO_*=0.0027; *P_20 min_-_male CTRL-female CTRL_*=0.9998; *P_20 min_-_male CKO-female CKO_*>0.9999; *P_30 min_-_male-CTRL-CKO_* =0.0155; *P_30 min_-_female CTRL-CKO_*=0.0414; *P_30 min_-_male CTRL-female CTRL_*=0.9525; *P_30 min_-_male CKO-female CKO_* =0.9965), ambulatory time (**H**: F_genotype_(1, 59)=29.24, *P***<**0.0001; F_sex_(1, 59)=0.0342, *P*=0.8538; F_intersection_(1, 59)=0.0048, *P*=0.9452; *P_male-CTRL-CKO_* =0.0024; *P_female CTRL-CKO_*=0.0015; *P_male CTRL-female CTRL_*>0.9999; *P_male CKO-female CKO_*>0.9999), and average velocity in the ambulatory time (**I**: F_genotype_(1, 59)=34.62, *P***<**0.0001; F_sex_(1, 59)=0.7594, *P*=0.3780; F_intersection_(1, 59)=1.611, *P*=0.2093; *P_male-CTRL-CKO_***<**0.0001; *P_female CTRL-CKO_*=0.0093; *P_male CTRL-female CTRL_*=0.4902; *P_male CKO-female CKO_*>0.9999) compared to relative control mice. N=21 in male Control group, N=11 in male Med23 CKO group, N=19 in female Control group, N=12 in female Med23 CKO group. Error bars in graphs represent means ± SEM. ***P*<0.01, ****P*<0.001, Two-way ANOVA with Bonferroni correction analysis for F, H and I, Three-way ANONA with Tukey correction analysis for G. **J** Both sexes of Med23 CKO mice showed impaired working memory shown by the SAB score of Y maze, compared to control mice with the same sex. N=22 in male Control group, N=13 in male Med23 CKO group, N=19 in female Control group, N=12 in female Med23 CKO group. F_genotype_(1, 62)=34.67, *P***<**0.0001; F_sex_(1, 62)=1.094, *P*=0.2996; F_intersection_(1, 62)=1.061, *P*=0.3071; *P_male-CTRL-CKO_***=**0.0047; *P_female CTRL-CKO_***<**0.0001; *P_male CTRL-female CTRL_*>0.9999; *P_male CKO-female CKO_*>0.9999. Error bars in graphs represent means ± SEM. ***P*<0.01, ****P*<0.001, Two-way ANOVA with Bonferroni correction analysis. **K** The values of prepulse inhibiton test at 3 levels of prepulse intensities of 65 (F_genotype_(1, 59)=20.64, *P***<**0.0001; F_sex_(1, 59)=1.415, *P*=0.2390; F_intersection_(1, 59)=0.2170, *P*=0.6431; *P_male-CTRL-CKO_***=**0.0049; *P_female CTRL-CKO_***=**0.0317; *P_male CTRL-female CTRL_*>0.9999; *P_male CKO-female CKO_*>0.9999), 73 (F_genotype_(1, 59)=28.56, *P***<**0.0001; F_sex_(1, 59)=0.2486, *P*=0.6199; F_intersection_(1, 59)=2.426, *P*=0.1246; *P_male-CTRL-CKO_***<**0.0001; *P_female CTRL-CKO_***=**0.0457; *P_male CTRL-female CTRL_*=0.5649; *P_male CKO-female CKO_*>0.9999), and 82 dB (F_genotype_(1, 59)=19.29, *P***<**0.0001; F_sex_(1, 59)=1.495, *P*=0.2263; F_intersection_(1, 59)=0.0001, *P*=0.9918; *P_male-CTRL-CKO_***=**0.0186; *P_female CTRL-CKO_***=**0.0164; *P_male CTRL-female CTRL_*>0.9999; *P_male CKO-female CKO_*>0.9999) in control and Med23 CKO mice. N=21 in male Control group, N=11 in male Med23 CKO group, N=19 in female Control group, N=12 in female Med23 CKO group. Error bars in graphs represent means ± SEM. ***P*<0.01, ****P*<0.001, Two-way ANOVA with Bonferroni correction analysis. **L-M** The recognition index for new object and familiar object of control and Med23 CKO mice in a 3-min retention session (**G**: F_genotype_(1, 50)=22.09, *P***<**0.0001; F_sex_(1, 50)=0.2092, *P*=0.6493; F_intersection_(1, 50)=0.5390, *P*=0.4663; *P_male-C-CKO_***=**0.0021; *P_female CTRL-CKO_***=**0.0423; *P_male CTRL-female CTRL_*>0.9999; *P_male CKO-female CKO_*>0.9999) or 5-min retention session (**H**: F_genotype_(1, 50)=18.19, *P***<**0.0001; F_sex_(1, 50)=1.185, *P*=0.2817; F_intersection_(1, 50)=0.1445, *P*=0.7054; *P_male-CTRL-CKO_***=**0.0114; *P_female CTRL-CKO_***=**0.0494; *P_male CTRL-female CTRL_*>0.9999; *P_male CKO-female CKO_*>0.9999). Both sexes of CKO mice exhibited inattentional phenotypes. N=19 in male Control group, N=9 in male Med23 CKO group, N=16 in female Control group, N=10 in female Med23 CKO group. Error bars in graphs represent means ± SEM. ***P*<0.01, ****P*<0.001, Two-way ANOVA with Bonferroni correction analysis. **N-P** The distance traveled during the CAR test (**I**: F_genotype_(1, 58)=13.25, *P*=0.0006; F_sex_(1, 58)=0.3463, *P*=0.5585; F_intersection_(1, 58)=0.7218, *P*=0.3991; *P_male-CTRL-CKO_***=**0.0156; *P_female CTRL-CKO_***=**0.062; *P_male CTRL-female CTRL_*>0.9999; *P_male CKO-female CKO_*>0.9999), the number of falls (**J**: F_genotype_(1, 58)=34.69, *P***<**0.0001; F_sex_(1, 58)=2.281, *P*=0.1364; F_intersection_(1, 58)=1.855, *P*=0.1784; *P_male-CTRL-CKO_***<**0.0001; *P_female CTRL-CKO_***=**0.0122; *P_male CTRL-female CTRL_*>0.9999; *P_male CKO-female CKO_*=0.4525), and the time course of incidence of control and Med23 CKO mice (**K**: Chi square=19.08, df=3, *P***=**0.0003). The impulsive behaviors occurred in both sexes of CKO mice. N=20 in male Control group, N=11 in male Med23 CKO group, N=19 in female Control group, N=12 in female Med23 CKO group. Error bars in graphs represent means ± SEM. **P*<0.05, ****P*<0.001, Two-way ANOVA with Bonferroni correction analysis for N and O, Log-rank (Mantel-Cox) test for P. CTRL: Control; CKO: Med23 CKO.


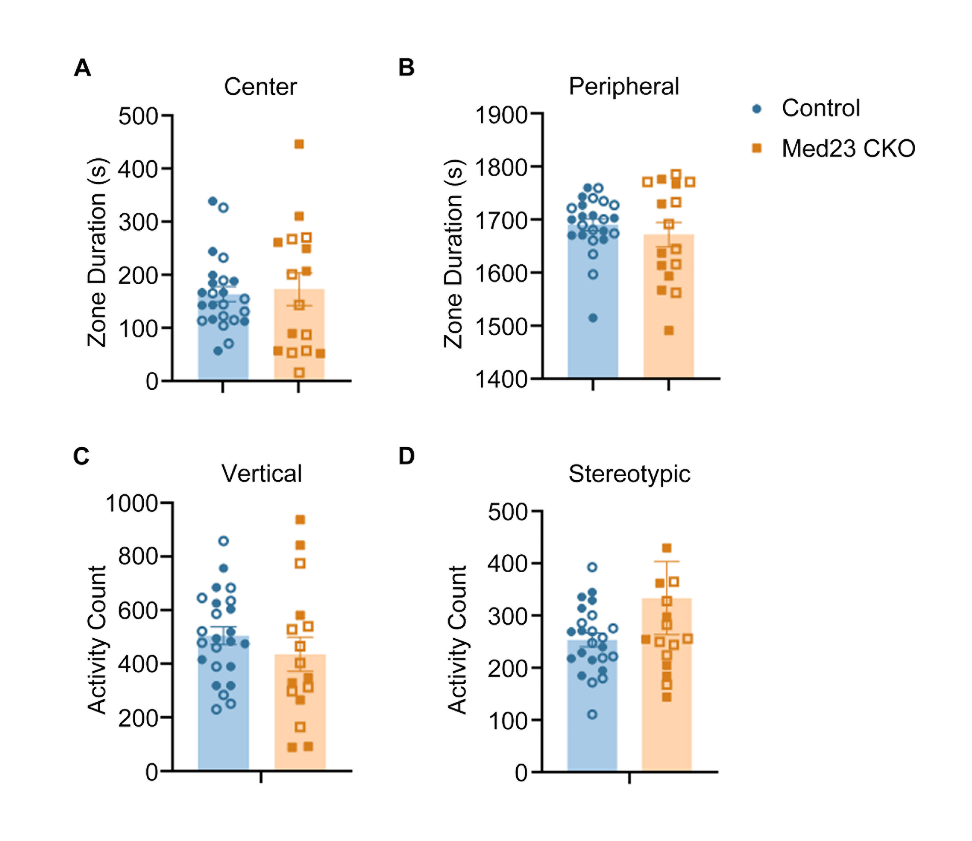


**Fig. S2: Med23 deficiency did not influence the anxiety level of mice in the open field test. A-B** The center (**A**: t=0.3091, df=38, *P*=0.7589) and peripheral zone duration (**B**: t=0.7934, df=38, *P*=0.4325) of control and Med23 CKO mice. **C-D** The vertical (**C**: t=1.049, df=38, *P*=0.3001) and stereotypic activity count (**D**: t=1.359, df=38, *P*=0.1821) of control and Med23 CKO mice. N=24 in control group, N=16 in Med23 CKO group. Error bars in graphs represent mean ± SEM. Two- tailed Student’s t test for A, B, C and D. Solid symbols represent male mice, and hollow symbols represent female mice.

**
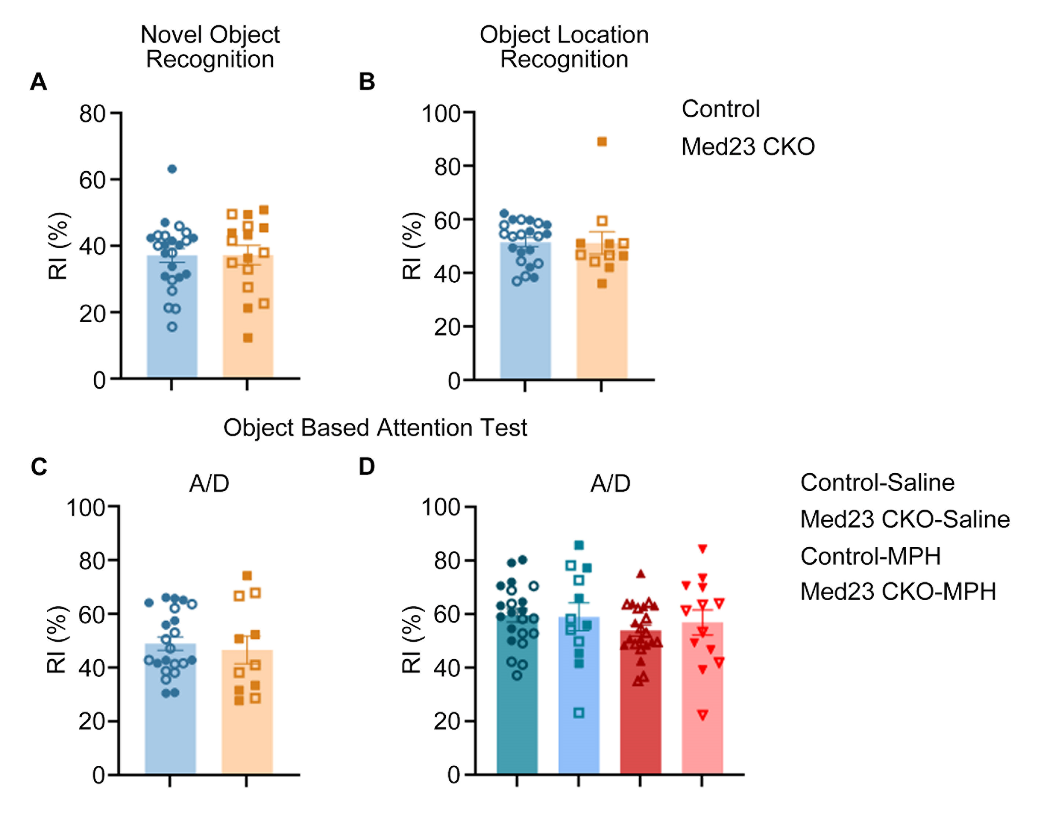
**

**Fig. S3: Med23 CKO mice exhibited intact abilities in object recognition and spatial learning.** **A** The recognition index of control and Med23 CKO mice in the novel object recognition test. N=24 in control group, N=16 in Med23 CKO group. t=0.0273, df=38, *P*=0.9784. Error bars in graphs represent mean ± SEM. Two- tailed Student’s t test. **B** The recognition index in the novel object recognition test of control and Med23 CKO mice. N=22 in control group, N=12 in Med23 CKO group. t=0.05635, df=13.28, P=0.9559. Error bars in graphs represent mean ± SEM. Two- tailed Student’s t test with Welch correction analysis. **C** The recognition index for object A and object D in the exploration session of control and Med23 CKO mice in the object-based attention test. N=22 in control group, N=11 in Med23 CKO group. t=0.4786, df=31, *P*=0.6356. Error bars in graphs represent mean ± SEM. Two- tailed Student’s t test. **D** The recognition index for object A and object D in the exploration session of control and Med23 CKO mice in the object-based attention test after saline or MPH treatment. N=22 in control-Saline group, N=12 in Med23 CKO-Saline group. N=22 in control-MPH group, N=13 in Med23 CKO-MPH group. F_genotype_(1, 65)=0.1291, *P*=0.7205; F_treatment_(1, 65)=0.1.339, *P*=0.2514; F_intersection_(1, 29)=0.2775, *P*=0.6001. Error bars in graphs represent mean ± SEM. Two-way ANOVA with Bonferroni correction analysis. Solid symbols represent male mice, and hollow symbols represent female mice.

**
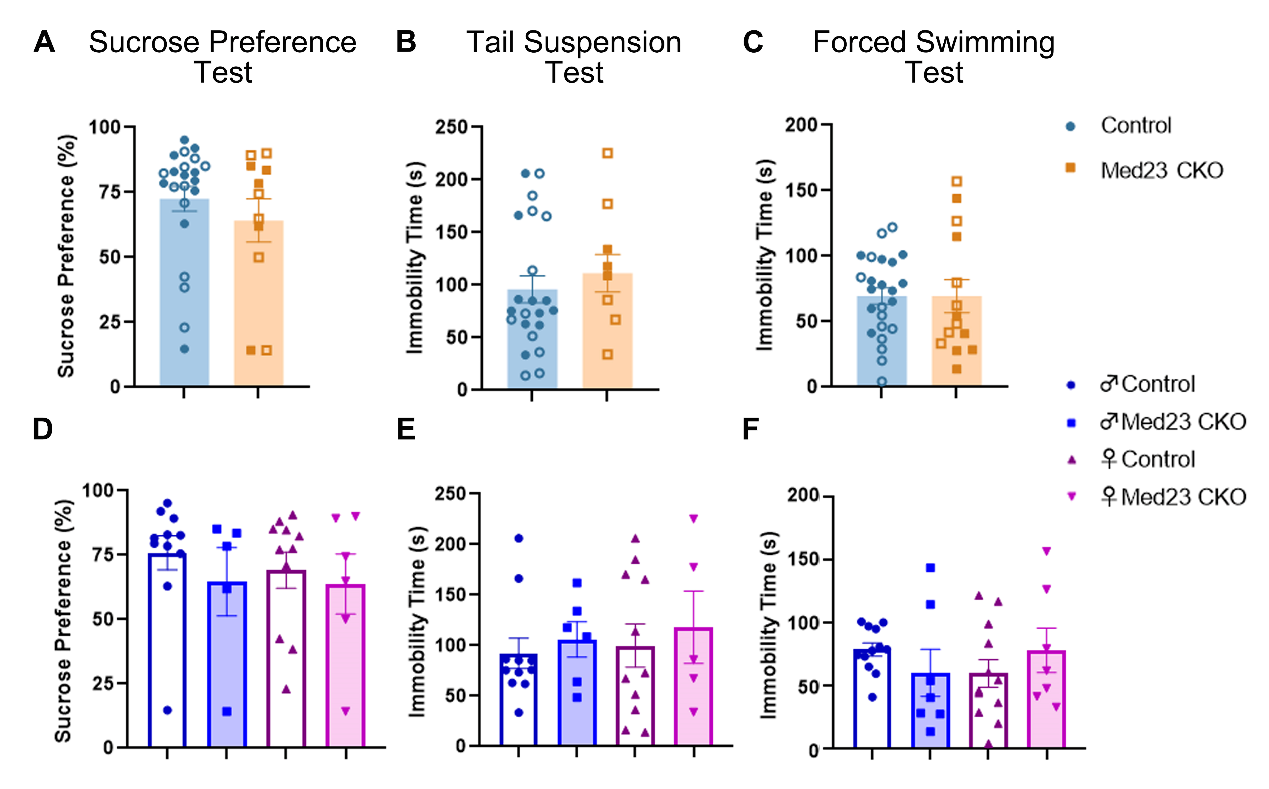
**

**Fig. S4: Med23 CKO mice exhibited intact depression-like behaviors. A** The sucrose preference of control and Med23 CKO mice. N=22 in control group, N=11 in Med23 CKO group. t=0.9365, df=31, *P*=0.3562. Error bars in graphs represent means ± SEM. Two- tailed Student’s t test. **B** The immobility time of control and Med23 CKO mice in tail suspension test. N=22 in control group, N=11 in Med23 CKO group. t=0.7454, df=31, *P*=0.4616. Error bars in graphs represent means ± SEM. Two- tailed Student’s t test. **C** The immobility time of control and Med23 CKO mice in forced swimming test. N=24 in control group, N=14 in Med23 CKO group. t=0.0006, df=36, *P*=0.9995. Error bars in graphs represent means ± SEM. Two- tailed Student’s t test. **D-F** Both sexes of Med23 CKO mice showed no obvious depression-like behaviors in the sucrose preference test (**D**: F_genotype_(1, 29)=0.8199, *P*=0.3727; F_sex_(1, 29)=0.1717, *P*=0.6817; F_intersection_(1, 29)=0.1033, *P*=0.7502), the tail suspension test (**E**: F_genotype_(1, 29)=0.4861, *P*=0.4912; F_sex_(1, 29)=0.1910, *P*=0.6653; F_intersection_(1, 29)=0.0085, *P*=0.9272) and the forced swimming test (**F**: F_genotype_(1, 34)=4.113×10^−7^, *P*=0.9995; F_sex_(1, 34)=0.0018, *P*=0.9559; F_intersection_(1, 34)=2.166, *P*=0.1503). Error bars in graphs represent means ± SEM. Two- tailed Student’s t test for A, B and C, two-way ANOVA with Bonferroni correction analysis for D, E and F. Solid symbols represent male mice, and hollow symbols represent female mice.

**
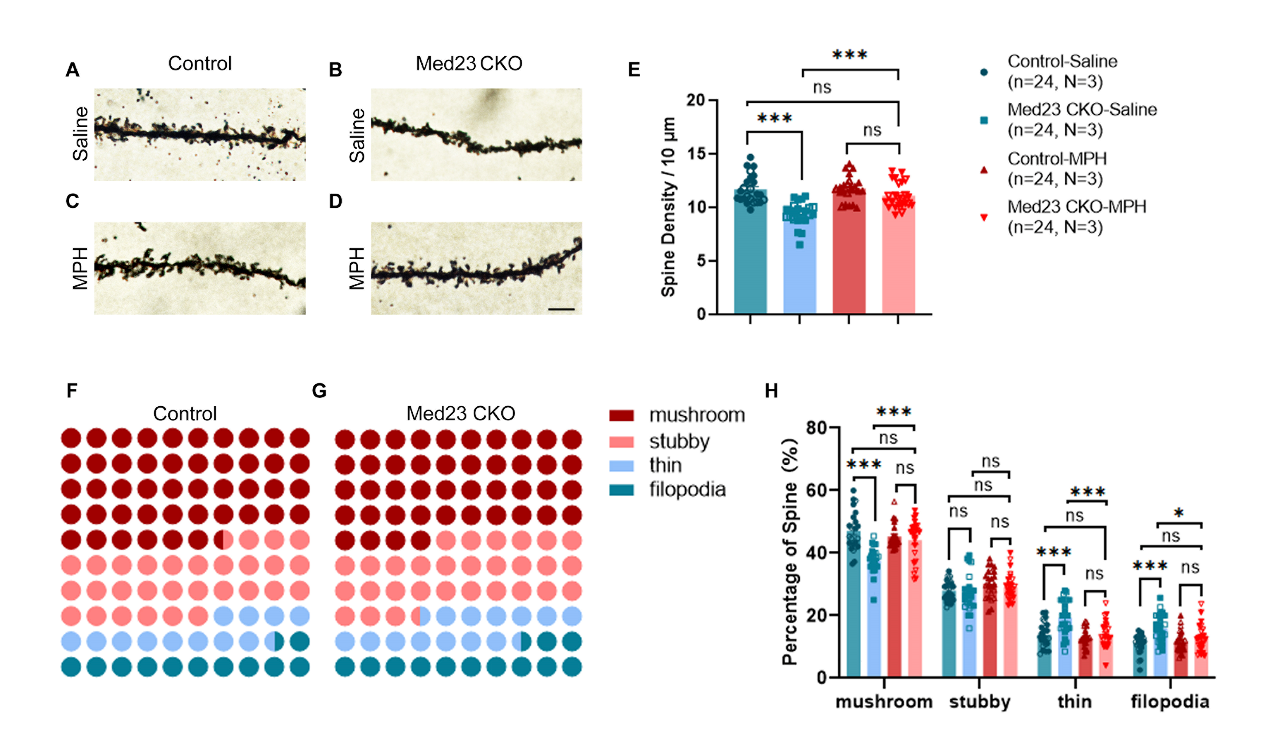
**

**Fig. S5: Chronic MPH administration restores deficits in the spines of granule cells after Med23 deletion. A**-**D** The representative images of spines from the secondary dendrites in DG granule cells for control, Med23 CKO after saline or chronic MPH administration. Scale bar, 5 µm. **E** The spine density of per 10 μm in control and Med23 CKO mice treated with saline or chronic MPH administration. n=24 dendrites from N=3 mice in control-saline group. n=24 dendrites from N=3 mice in Med23 CKO-saline group. n=24 dendrites from N=3 mice in control-MPH group. n=24 dendrites from N=3 mice in Med23 CKO-MPH group. F_genotype_(1, 92)=37.13, *P*<0.0001; F_treatment_(1, 92)=13.52, *P*=0.0004; F_intersection_(1, 92)=11.18, *P*=0.0012; *P_CTRL-SAL-CKO-SAL_*<0.0001; *P_CTRL-SAL-CKO-MPH_*=0.5457; *P_CKO-SAL-CKO-MPH_*<0.0001; *P_CTRL-MPH-CKO-MPH_*=0.3296. ****P*<0.001. Error bars in graphs represent mean ± SEM. Two-way ANOVA with Bonferroni correction analysis. **F**-**G** The dot plot graphs for the percentage of four spine categories in control and Med23 CKO mice with 2-week MPH treatment. **H** The percentage of mushroom- (F_genotype_(1, 92)=23.50, *P*<0.0001; F_treatment_(1, 92)=4.585, *P*=0.0349; F_intersection_(1, 92)=13.99, *P*=0.0003; *P_CTRL-SAL-CKO-SAL_*<0.0001; *P_CTRL-SAL-CKO-MPH_*=0.3523; *P_CKO-SAL-CKO-MPH_*=0.0004; *P_CTRL-MPH-CKO-MPH_*>0.9999), stuby- (F_genotype_(1, 92)=0.9692, *P*=0.3274; F_treatment_(1, 92)=5.234, *P*=0.0244; F_intersection_(1, 92)=0.0048, *P*=0.9445; *P_CTRL-SAL-CKO-SAL_*>0.9999; *P_CTRL-SAL-CKO-MPH_*>0.9999; *P_CKO-SAL-CKO-MPH_*=0.7213; *P_CTRL-MPH-CKO-MPH_*>0.9999), thin- (F_genotype_(1, 92)=13.26, *P*=0.0004; F_treatment_(1, 92)=13.71, *P*=0.0004; F_intersection_(1, 92)=5.397, *P*=0.0224; *P_CTRL-SAL-CKO-SAL_*=0.0003; *P_CTRL-SAL-CKO-MPH_*>0.9999; *P_CKO-SAL-CKO-MPH_*=0.0003; *P_CTRL-MPH-CKO-MPH_*>0.9999) and filopodia-shaped (F_genotype_(1, 92)=14.30, *P*=0.0003; F_treatment_(1, 92)=2.399, *P*=0.1248; F_intersection_(1, 92)=6.089, *P*=0.0154; *P_CTRL-SAL-CKO-SAL_*=0.0002; *P_CTRL-SAL-CKO-MPH_*>0.9999; *P_CKO-SAL-CKO-MPH_*=0.0333; *P_CTRL-MPH-CKO-MPH_*>0.9999) spines in four groups. n=24 dendrites from N=3 mice in control-saline group. n=24 dendrites from N=3 mice in Med23 CKO-saline group. n=24 dendrites from N=3 mice in control-MPH group. n=24 dendrites from N=3 mice in Med23 CKO-MPH group. Error bars in graphs represent mean ± SEM. Two-way ANOVA with Bonferroni correction analysis. Solid symbols represent male mice, and hollow symbols represent female mice. CTRL-SAL: Control-Saline group; CTRL-MPH: Control-MPH group; CKO-SAL: Med23 CKO-Saline group; CKO-MPH: Med23 CKO-MPH group.

**
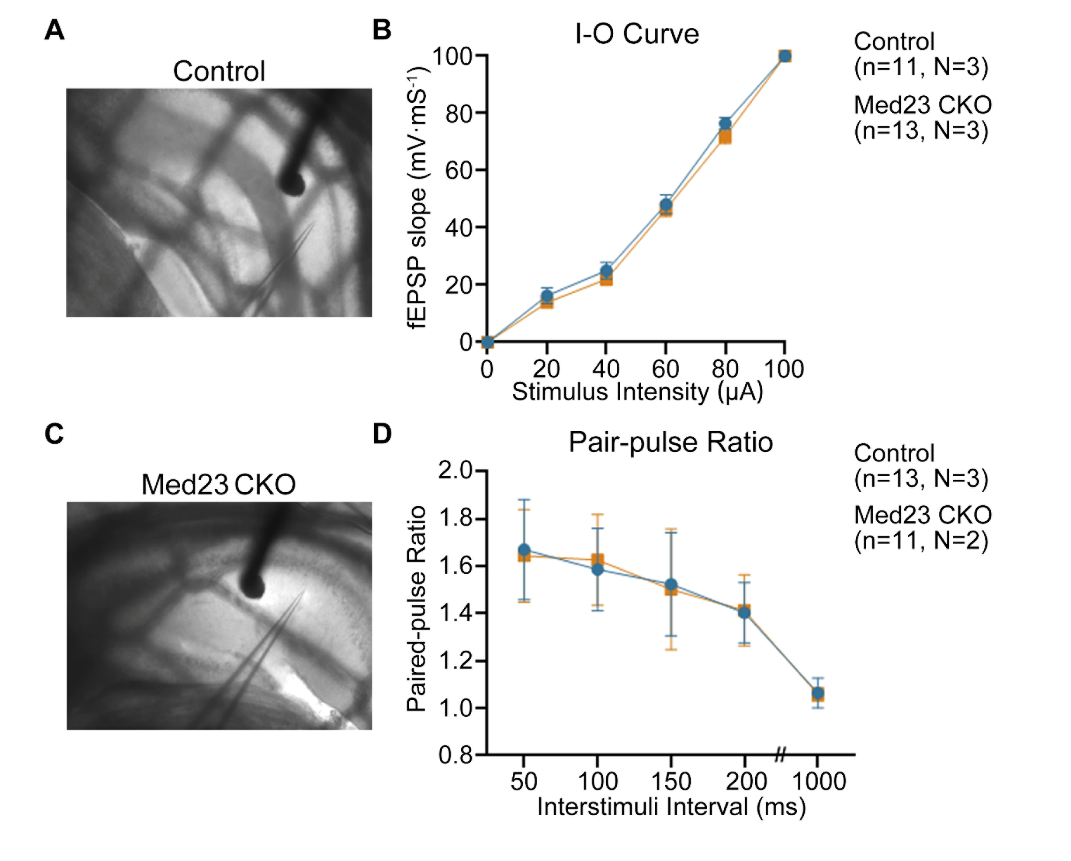
**

**Fig. S6: The basic synaptic transmission and short-term plasticity was not impaired in CA3-CA1 pathway in Med23 CKO mice. A**, **C** Representative images of the recorded areas (the stratum radiatum of the dorsal CA1) in control and Med23 CKO mice. **B** The I-O curves for input-output recordings plotted with stimulus intensity against slope showed no difference in control and Med23 CKO mice. n=5 from N=1 mice in control group, n=13 from N=3 mice in Med23 CKO group. F_genotype_(1, 132)=2.689, *P*=0.1034; F_intensity_(5, 132)=696.0, *P*<0.0001; F_intersection_(5, 132)=0.3779, *P*=0.8632. Error bars in graphs represent means ± SEM. Two-way ANOVA with Bonferroni correction analysis. **D** The PPR for short-term synaptic plasticity was similar in control and Med23 CKO mice. n=6 from N=1 mice in control group, n=13 from N=3 mice in Med23 CKO group. Data were shown as means ± SEM. One-way ANOVA with Tukey correction analysis. F_genotype_(1, 110)=0.0008, *P*=0.9770; F_interval_(4, 110)=43.68, *P*<0.0001; F_intersection_(4, 110)=0.1425, *P*=0.9659. Error bars in graphs represent means ± SEM. Two-way ANOVA with Bonferroni correction analysis.

**Reference**

1. Zhang J-B, Chen L, Lv Z-M, Niu X-Y, Shao C-C, Zhang C, et al. Oxytocin is implicated in social memory deficits induced by early sensory deprivation in mice. Mol Brain. 2016;9:98.

2. Denninger JK, Smith BM, Kirby ED. Novel Object Recognition and Object Location Behavioral Testing in Mice on a Budget. J Vis Exp. 2018:10.3791/58593.

3. Hu Y-Q, Niu T-T, Xu J-M, Peng L, Sun Q-H, Huang Y, et al. Negative air ion exposure ameliorates depression-like behaviors induced by chronic mild stress in mice. Environ Sci Pollut Res Int. 2022;29:62626–62636.
